# A Risk Model Developed Based on Homologous Recombination Deficiency Predicts Overall Survival in Patients With Lower Grade Glioma

**DOI:** 10.3389/fgene.2022.919391

**Published:** 2022-07-01

**Authors:** Hao Peng, Yibiao Wang, Pengcheng Wang, Chuixue Huang, Zhaohui Liu, Changwu Wu

**Affiliations:** ^1^ Department of Neurosurgery, Hainan General Hospital, Haikou, China; ^2^ Department of Neurosurgery, The Second People’s Hospital of Hainan Province, Wuzhishan, China; ^3^ Institute of Anatomy, University of Leipzig, Leipzig, Germany; ^4^ Department of Neurosurgery, Xiangya Hospital, Central-South University, Changsha, China

**Keywords:** homologous recombination deficiency, lower grade glioma, prognosis, risk model, immune cell infiltration

## Abstract

The role of homologous recombination deficiency (HRD) in lower grade glioma (LGG) has not been elucidated, and accurate prognostic prediction is also important for the treatment and management of LGG. The aim of this study was to construct an HRD-based risk model and to explore the immunological and molecular characteristics of this risk model. The HRD score threshold = 10 was determined from 506 LGG samples in The Cancer Genome Atlas cohort using the best cut-off value, and patients with high HRD scores had worse overall survival. A total of 251 HRD-related genes were identified by analyzing differentially expressed genes, 182 of which were associated with survival. A risk score model based on HRD-related genes was constructed using univariate Cox regression, least absolute shrinkage and selection operator regression, and stepwise regression, and patients were divided into high- and low-risk groups using the median risk score. High-risk patients had significantly worse overall survival than low-risk patients. The risk model had excellent predictive performance for overall survival in LGG and was found to be an independent risk factor. The prognostic value of the risk model was validated using an independent cohort. In addition, the risk score was associated with tumor mutation burden and immune cell infiltration in LGG. High-risk patients had higher HRD scores and “hot” tumor immune microenvironment, which could benefit from poly-ADP-ribose polymerase inhibitors and immune checkpoint inhibitors. Overall, this big data study determined the threshold of HRD score in LGG, identified HRD-related genes, developed a risk model based on HRD-related genes, and determined the molecular and immunological characteristics of the risk model. This provides potential new targets for future targeted therapies and facilitates the development of individualized immunotherapy to improve prognosis.

## Introduction

World Health Organization (WHO) grade II and III gliomas are considered lower grade gliomas (LGGs), which have a slower course as well as a better prognosis compared to glioblastoma (GBM, grade IV) (Jiang et al., 2016; Lapointe et al., 2018). Unfortunately, recurrence and malignant progression of LGG are almost unavoidable, even with comprehensive treatments, including surgical resection, radiotherapy, and chemotherapy (Comprehensive, 2015). This may be due to the limited treatment options and treatment resistance in LGG (Comprehensive, 2015; Wu et al., 2020). In addition, there is a wide range of survival (from 1 to 15 years) in LGG (Comprehensive, 2015), and a growing number of studies have shown that even with similar grades, patients with LGG differ greatly in clinical outcomes (Xiao et al., 2020). However, traditional methods based on histopathological classification are not sufficient to predict clinical outcomes (Van Den Bent, 2010). Therefore, clinicians and oncologists are increasingly inclined to use genetic testing to predict prognosis and guide clinical decisions (Theeler et al., 2012; Appin and Brat, 2014; Eckel-Passow et al., 2015). Currently, several biomarkers, including isocitrate dehydrogenase 1 (IDH1) (Kwon et al., 2020) and O6-methylguanine DNA methyltransferase (MGMT) (Binabaj et al., 2018), have become important markers of LGG clinical behavior and are closely associated with prognosis. To gain additional insights, further development of prognostic markers for LGG is needed to facilitate individualized treatment and provide additional potential therapeutic targets.

Recently, poly ADP-ribose polymerase (PARP) inhibitors were approved for use by the Food and Drug Administration and recommended for the treatment of tumors with BRCA1/2 mutations, including pancreatic cancer and prostate cancer (Rescigno et al., 2018; Li et al., 2021; Zhuang et al., 2021). Tumors with BRCA1/2 mutations are often accompanied by homologous recombination deficiency (HRD), and cancer cells with HRD are more sensitive to PARP inhibitors (Rescigno et al., 2018; Chen et al., 2021). Extensive studies in gliomas have demonstrated the radiosensitizing and chemosensitizing properties of PARP inhibitors (Gupta et al., 2014; Lesueur et al., 2017; Gupta et al., 2019). In a recent study, increased homologous recombination made glioma cells resistant to temozolomide (TMZ), while homologous recombination inhibition re-sensitized resistant cells, demonstrating that HRD cells are more sensitive to TMZ (Ohba et al., 2021). The HRD score developed based on genomic scars was designed to quantify HRD and has now been applied to breast cancer (Telli et al., 2016), prostate cancer (Sztupinszki et al., 2020), and ovarian cancer (Davies et al., 2017) with the same threshold. However, due to the great heterogeneity among different tumor types, it could be more rational to use different thresholds for classification in different tumor types (Jonsson et al., 2019). In addition, because HRD is a genomic event, its changes can be reflected by transcriptome level assays (Peng et al., 2014; Ladan et al., 2021; Zhuang et al., 2021), and changes in the transcriptome can also provide new insights into the changes in HRD. However, the threshold of HRD score in LGG is not known and the transcriptomic features of HRD in LGG have not been fully investigated.

In this study, we explored the role of HRD in LGG, determined the threshold of HRD, and identified HRD-related genes based on transcriptome sequencing. Importantly, we explored the prognostic role of HRD-related genes in LGG and constructed a risk model that not only effectively predicted prognosis, but also distinguished different immunological and molecular features.

## Materials and Methods

### Patients and Datasets

Normalized RNA-seq data and clinical information for 506 LGG samples from The Cancer Genome Atlas (TCGA) cohort and 431 LGG samples from the Chinese Glioma Genome Atlas (CGGA) cohort were obtained from GlioVis (http://gliovis.bioinfo.cnio.es/) (Bowman et al., 2017). In addition, we also obtained a microarray cohort (Rembrandt cohort) containing 141 LGG samples from GlioVis. The RNA-seq and microarray data were log_2_(x + 0.5) transformed. Only samples with complete survival information were included in this study. Somatic mutation counts, microsatellite instability (MSI)-sensor scores, aneuploidy scores, and fraction genome altered scores were obtained from the cBioPortal database (http://www.cbioportal.org). The VarScan-processed mutation dataset in the TCGA cohort was obtained from the Genomic Data Commons Data Portal (https://portal.gdc.cancer.gov/). The basic clinical information of all RNA-seq samples included in this study is summarized in Supplementary Table S1.

### Homologous Recombination Deficiency Score Analysis

The HRD score was defined as the unweighted sum of loss of heterozygosity (LOH), telomeric allelic imbalance (TAI), and large-scale state transition (LST) scores (Sztupinszki et al., 2018; Takaya et al., 2020). LOH (Abkevich et al., 2012), TAI (Birkbak et al., 2012), and LSL (Manié et al., 2016) were defined according to previous studies. The HRD score can be obtained from a pan-cancer study by Thorsson et al. (2018). The HRD scores of individual patients are summarized in Supplementary Table S2.

### Identification of Homologous Recombination Deficiency-Related Genes

First, we searched for the optimal threshold of HRD score based on “survminer” and “survival” R packages to classify LGG into high and low HRD score groups. Subsequently, we used the R package “limma” to obtain differentially expressed genes (DEGs) between the high and low HRD score groups using the threshold of log_2_|fold change (FC)| >1 and adjusted *p* value < 0.05, and these DEGs were defined as HRD-related genes.

### Construction of the Risk Model

First, we assessed the prognostic role of each HRD-related gene in LGG based on univariate Cox regression analysis, and prognostic-related genes were screened at a threshold of *p* < 0.05. Subsequently, the prognosis-related genes were further downscaled using least absolute shrinkage and selection operator (LASSO)-Cox regression analysis (Tibshirani, 1997). Finally, the genes obtained from the LASSO-Cox regression analysis were entered into a stepwise regression analysis to obtain the best risk model. The risk model was calculated using the following formula:

$$Risk\;score=\sum \beta_{i}\times \;Exp_{i}$$

where *β*_i_ is the coefficient of each gene in the final risk model and Exp_i_ is the gene expression value.

### Functional and Pathway Enrichment Analysis

Gene Ontology (GO) enrichment analysis and Kyoto Encyclopedia of Genes and Genomes (KEGG) pathway analysis of HRD-related genes were performed using the R package “clusterProfiler” (Yu et al., 2012). In addition, we assessed the biological processes and pathways enriched in the high-and high-risk score groups using gene set enrichment analysis (GSEA) based on the KEGG and HALLMARI gene sets from the MSigDB database (Subramanian et al., 2005). Adjusted *p* values < 0.25 were considered statistically significant. In addition, to explore the relevance of the risk model to immune-related biological processes, we obtained an immune activation-related gene set, an immune checkpoint-related gene set, and the T transforming growth factor (TGF)β/epithelial-mesenchymal transition (EMT) pathway-relevant gene set from a study by Zeng et al. (2019). Furthermore, a total of 18 important gene signatures, including CD8 T-effector signature and pan-fibroblast TGFβ response signature (Pan-F-TBRS) were obtained from Mariathasan et al. (2018) to explore the correlation between the risk model and other known core biological processes.

### Immune Cell Infiltration Analysis

Based on the R package “GSVA,” we assessed the level of immune cell infiltration in each sample using single-sample gene set enrichment analysis (ssGSEA). A total of 28 immune cells from previous studies were included in this study (Charoentong et al., 2017). In addition, the ImmuneScore, StromalScore, and ESTIMATEScore were calculated for each sample using the ESTAMATE algorithm (Yoshihara et al., 2013) (Supplementary File S1).

### Statistical Analysis

Differences between the two groups were compared using the Wilcoxon rank sum test. The correlation between the two variables was explored using Spearman’s correlation analysis. The R package “pROC” was used to plot receiver operating characteristic (ROC) curves to verify the validity of the model and obtain the area under the curve (AUC). The R package “survival” was used for Kaplan-Meier (KM) curve analysis and univariate and multivariate Cox regression analyses. All statistical analyses were performed using R software (Version 4.1.1). Statistical significance was set at *p* < 0.05, and unless otherwise stated and *p* values were two-sided.

## Results

### Association of Homologous Recombination Deficiency Score With Prognosis and Genomic Instability in Lower Grade Glioma Patients

**FIGURE 1:**
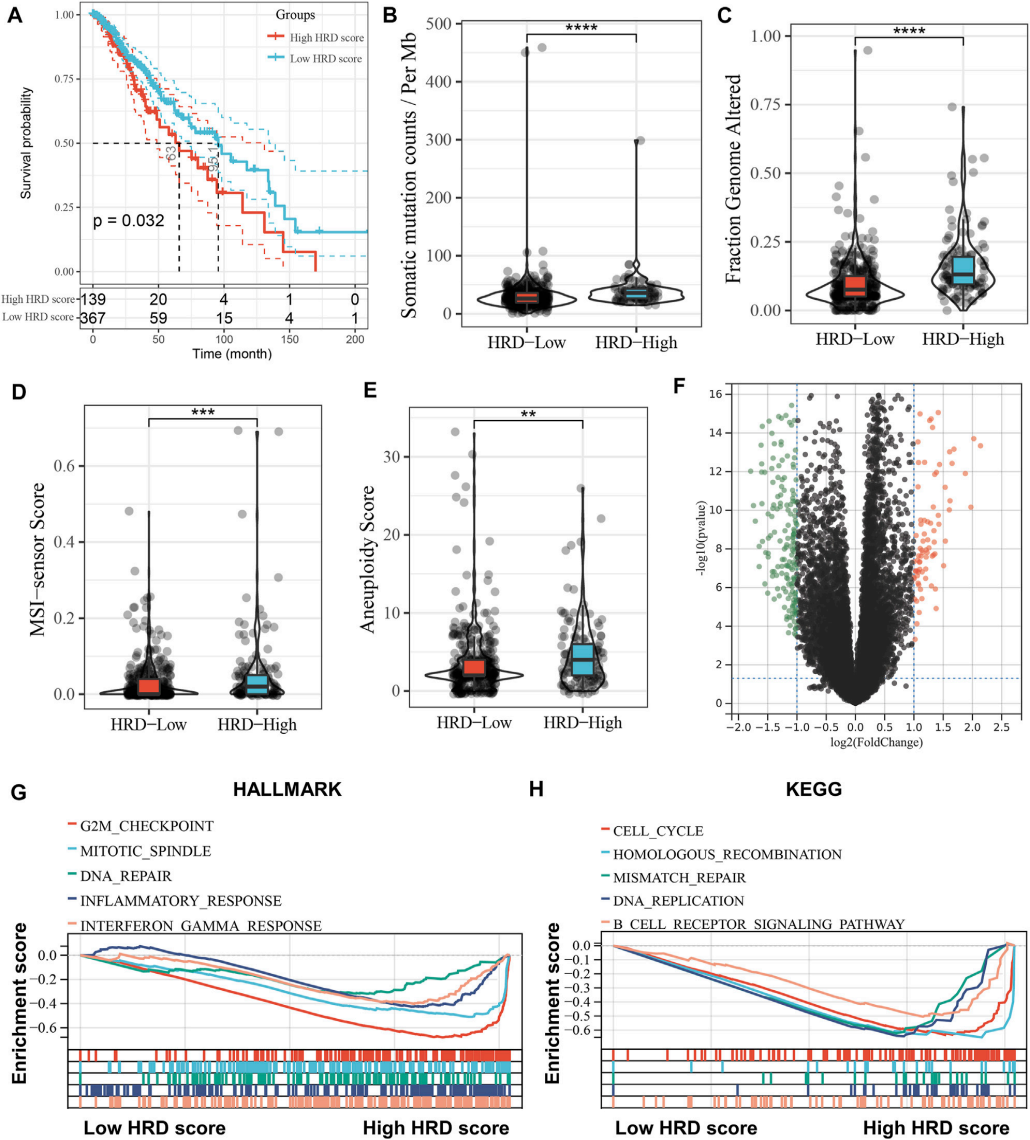
Association of HRD score with prognosis and genomic instability in LGG patients. **(A)**, Kaplan-Meier curve depicts the survival difference between high HRD score (HRD score > 10) and low HRD score (HRD score ≤ 10) groups (log-rank *p* = 0.032), with high and low HRD groups grouped by the best cut-off value. Red represents the high HRD group and blue represents the low HRD group. **(B–E)**, Violin plots of the differences in somatic mutation counts **(B)**, fraction genome altered **(C)**, MSI-sensor scores **(D)** and aneuploidy scores **(E)** between the high HRD and low HRD groups. **p < 0.01, ***p < 0.001, ****p < 0.0001. **(F)**, Volcano plot of differentially expressed genes in the high HRD group relative to the low HRD group. Red dots represent up-regulated genes (*n* = 84) and green represents down-regulated genes (*n* = 167). **(G,H)**, GSEA enrichment plots base on HALLMARK **(G)** and KEGG **(H)** gene sets showing the relatively significantly enriched pathways in high HRD score group.

After obtaining the HRD scores based on LOH, TAI, and LSL, we divided the LGG patients into high (HRD score > 10) and low HRD (HRD score ≤ 10) groups using the HRD score = 10 as the best cut-off value. Survival analysis showed that patients in the high HRD group had worse overall survival (OS) than those in the low HRD group (*p* = 0.032). Further, we explored the relationship between HRD scores and other genomic instability markers. As shown in Figures 1B–E, the high HRD score group had significantly higher somatic mutation counts (*p* < 0.0001), fraction genome altered (*p* < 0.0001), MSI-sensor scores (*p* < 0.001), and aneuploidy scores (*p* < 0.01) compared to the low HRD score group. GSEA analysis revealed that the high HRD score group was mainly enriched in genomic-related pathways such as homologous recombination, cell cycle, and DNA repair (Figures 1G,H). Interestingly, we also found that the high HRD score group was enriched in immune response-related pathways such as inflammatory response and interferon-gamma (IFNγ) response (Figures 1G,H). In addition, as shown in the volcano plot (Figure 1F), we identified 251 HRD-related genes by comparing the DEGs between the high and low HRD score groups, with 84 upregulated and 167 downregulated genes in the high HRD score group compared to the low HRD score group (Supplementary Table S3). GO and KEGG analyses showed that the HRD-related genes were mainly enriched in genome-related biological processes and pathways, such as chromatin separation, DNA binding, and cell cycle (Supplementary Figure S1).

### Construction of the Risk Model

**FIGURE 2:**
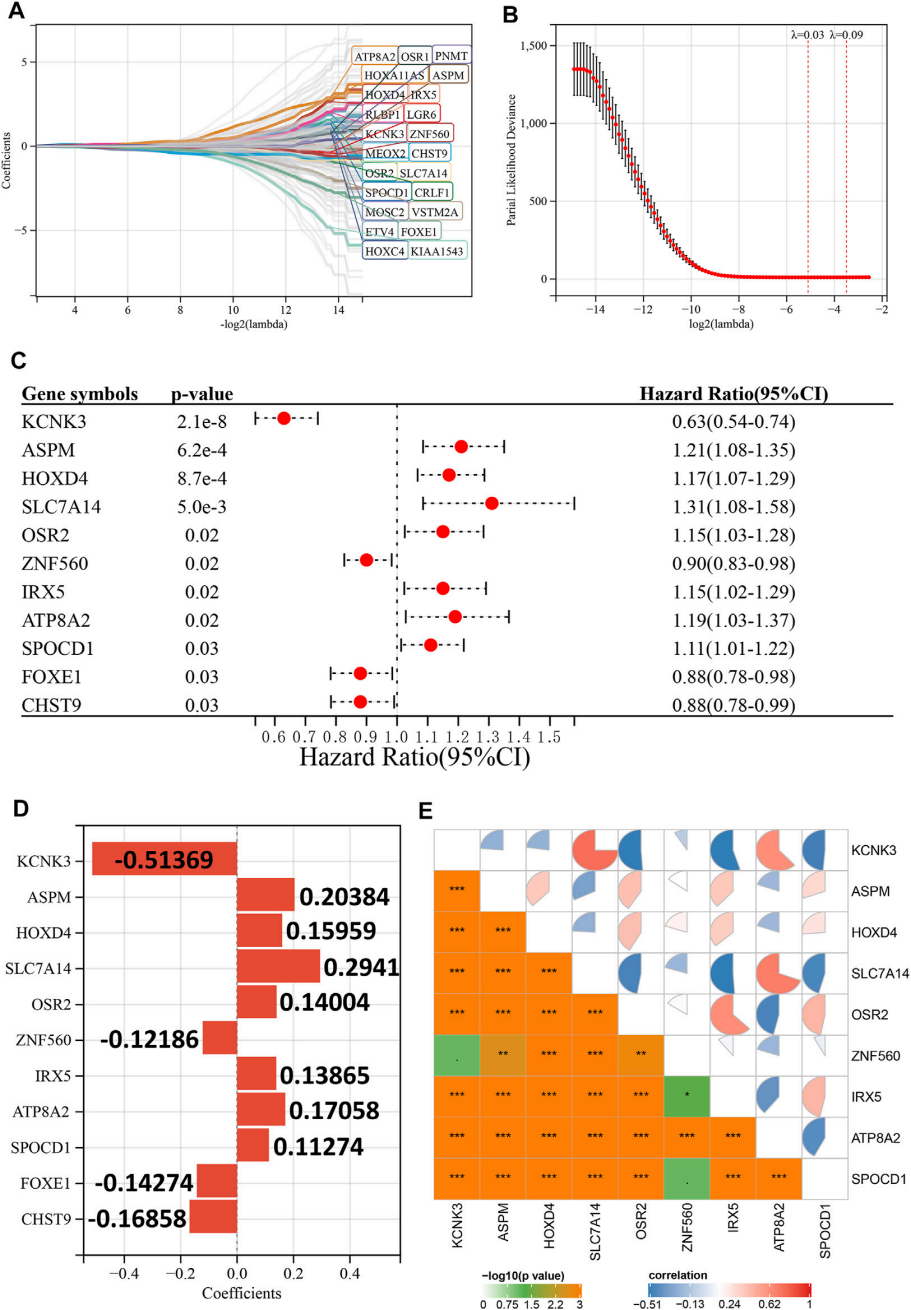
Construction of the risk model. **(A)**, LASSO coefficient profiles of 23 prognostic HRD-related genes. **(B)**, Ten-time cross-validation for tuning parameter selection in the LASSO model. **(C)**, Univariate Cox regression analysis reveals the association of 11 genes in the risk model with the prognosis of LGG. **(D)**, Coefficients for the 11 genes in the risk model. **(E)**, Expression correlation between 11 genes in the risk model. *p* < 0.1, *p < 0.05, **p < 0.01, ***p < 0.001.

First, we explored the prognostic value of HRD-related genes in LGG using univariate Cox regression analysis, and it is noteworthy that most HRD-related genes were associated with prognosis in LGG (182 out of 251). (Supplementary Table S4). Subsequently, we further screened 23 prognosis-related genes using LASSO-Cox regression analysis (Figures 2A,B), and finally constructed the optimal risk model using stepwise regression. This risk model had the largest C-index (C-index = 0.873) and contained 11 key HRD-related genes (KCNK3, ASPM, HOXD4, SLC7A14, OSR2, ZNF560, IRX5, ATP8A2, SPOCD1, FOXE1, and CHST9), of which four were favorable prognostic factors for LGG and seven were unfavorable prognostic factors (Figure 2C). The coefficients of each gene in the risk model are shown in Figure 2D, and it is noteworthy that the expression of the genes in the risk model possesses a wide range of correlations with each other (Figure 2E). In the Rembrandt cohort, we excluded ZNF560 and SPOCD1 due to the lack of expression data for these two genes to construct the risk model.

### The Association Between Risk Score and Prognosis of Lower Grade Glioma

**FIGURE 3:**
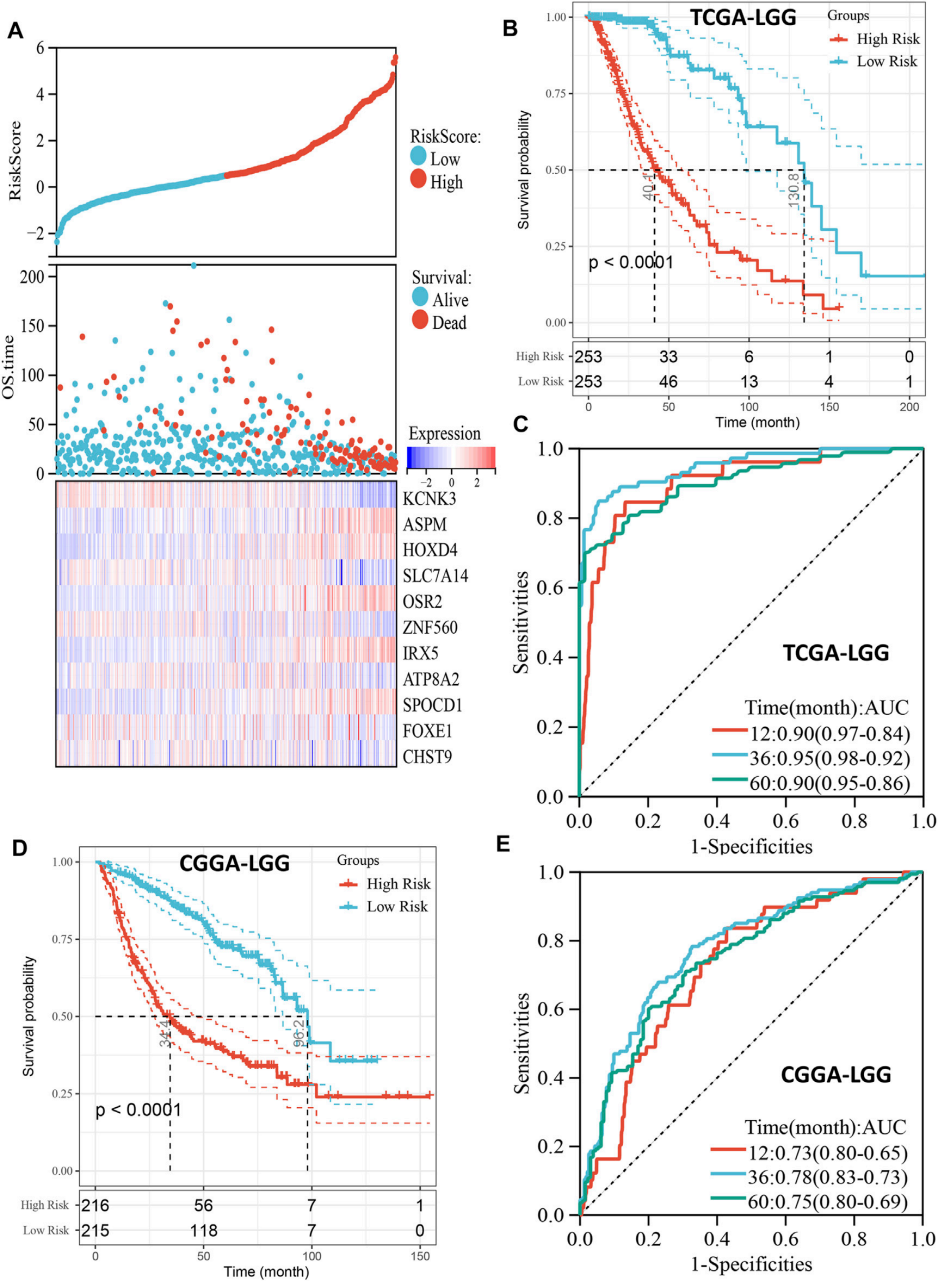
Prognostic predictive role of risk score. **(A)**, The distribution of risk score and survival status and the heatmap of gene expression of the risk model in TCGA cohort. **(B)**, Kaplan-Meier curve depicts the survival difference between high-risk and low-risk groups (log-rank *p* < 0.0001) in the TCGA cohort. Red representing the high-risk group and blue representing the low-risk group. **(C)**, ROC curve analysis of the risk score in the TCGA cohort. **(D)**, Kaplan-Meier curve depicts the survival difference between high-risk and low-risk groups (log-rank *p* < 0.0001) in the CGGA cohort. Red representing the high-risk group and blue representing the low-risk group. **(E)**, ROC curve analysis of the risk score in the CGGA cohort.

**FIGURE 4:**
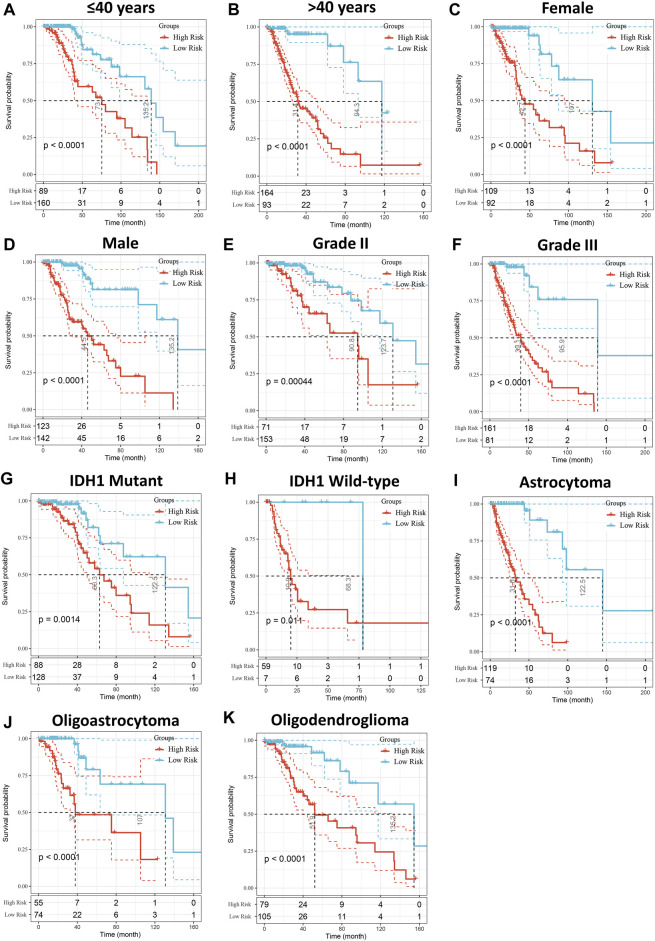
Stratified OS analysis based on the risk model in the TCGA cohort. Based on the risk score model, stratified OS analysis performed in patients with different clinical parameters, such as age **(A,B)**, gender **(C,D)**, WHO grade **(E,F)**, IDH1 status **(G,H)**, and histological type **(I–K)** in the TCGA cohort. Significance for survival analysis was calculated using a log-rank test, with the red line representing the high-risk group and the blue line representing the low-risk group. The grouping of LGG samples is shown at the bottom of the charts.

Patients with LGG in the TCGA cohort were divided into high- and low-risk groups based on the median of risk scores, and the high-risk group had more deaths than the low-risk group (Figure 3A). In addition, the high-risk score group also had significantly worse OS than the low-risk score group (*p* < 0.001, Figure 3B). The ROC curve indicated that the risk model had excellent predictive performance, and the AUCs of 1-, 3-, and 5-year OS were 0.90, 0.95, and 0.90, respectively (Figure 3C). Importantly, the predictive ability of our risk model for LGG prognosis was validated in the CGGA cohort (Figures 3D,E). It is worth noting that the risk model also has good predictive performance in Rembrandt cohort (Supplementary Figure S2). To further confirm the robustness of the risk model, we performed a data stratification analysis according to the different clinical characteristics of LGG patients. As shown in Figures 4A–K, patients with high-risk scores always had worse OS than patients with low-risk scores in subgroups with different age, gender, WHO grade, IDH1 status, and histological subtype in the TCGA cohort. In addition, the excellent performance of our risk scores in the stratification analysis was also verified in the CGGA cohort (Supplementary Figure S3).

**FIGURE 5:**
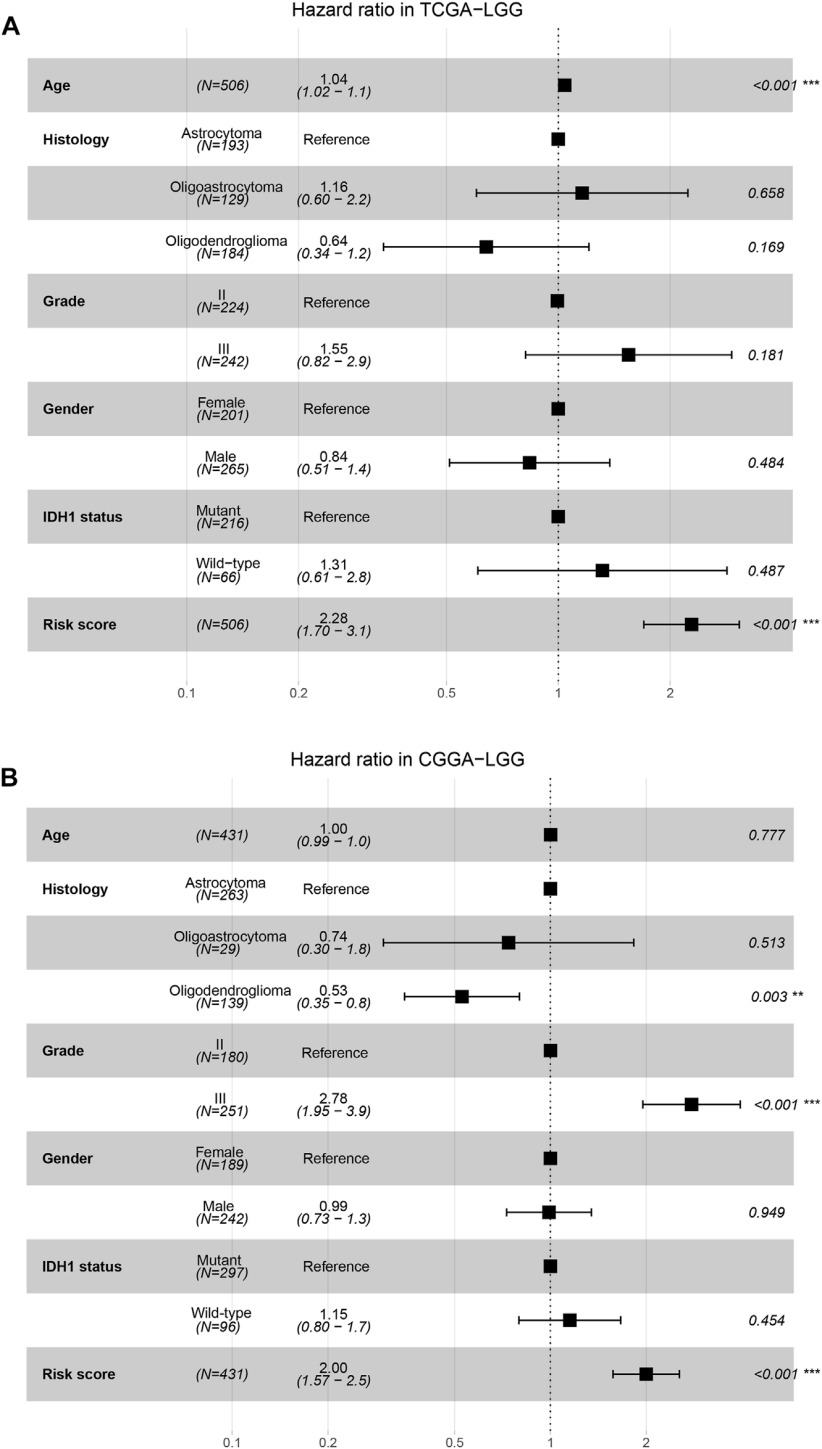
Independence of risk score as a risk factor. **(A,B)**, The forest plots show the multivariate Cox regression analysis using age, histological subtype, WHO grade, gender, IDH1 status and risk score as covariates in the TCGA **(A)** and CGGA **(B)** cohorts.

Univariate Cox regression analysis showed that the risk score was a prognostic factor in both the TCGA and CGGA cohorts (Supplementary Table S5). In the multivariate Cox regression analysis adjusted for age, histological subtype, WHO grade, gender, and IDH1 status as covariates, the risk score was also an independent prognostic factor in both TCGA and CGGA cohorts (Figures 5A,B).

### Clinical and Mutational Characteristics of Risk Score

**FIGURE 6:**
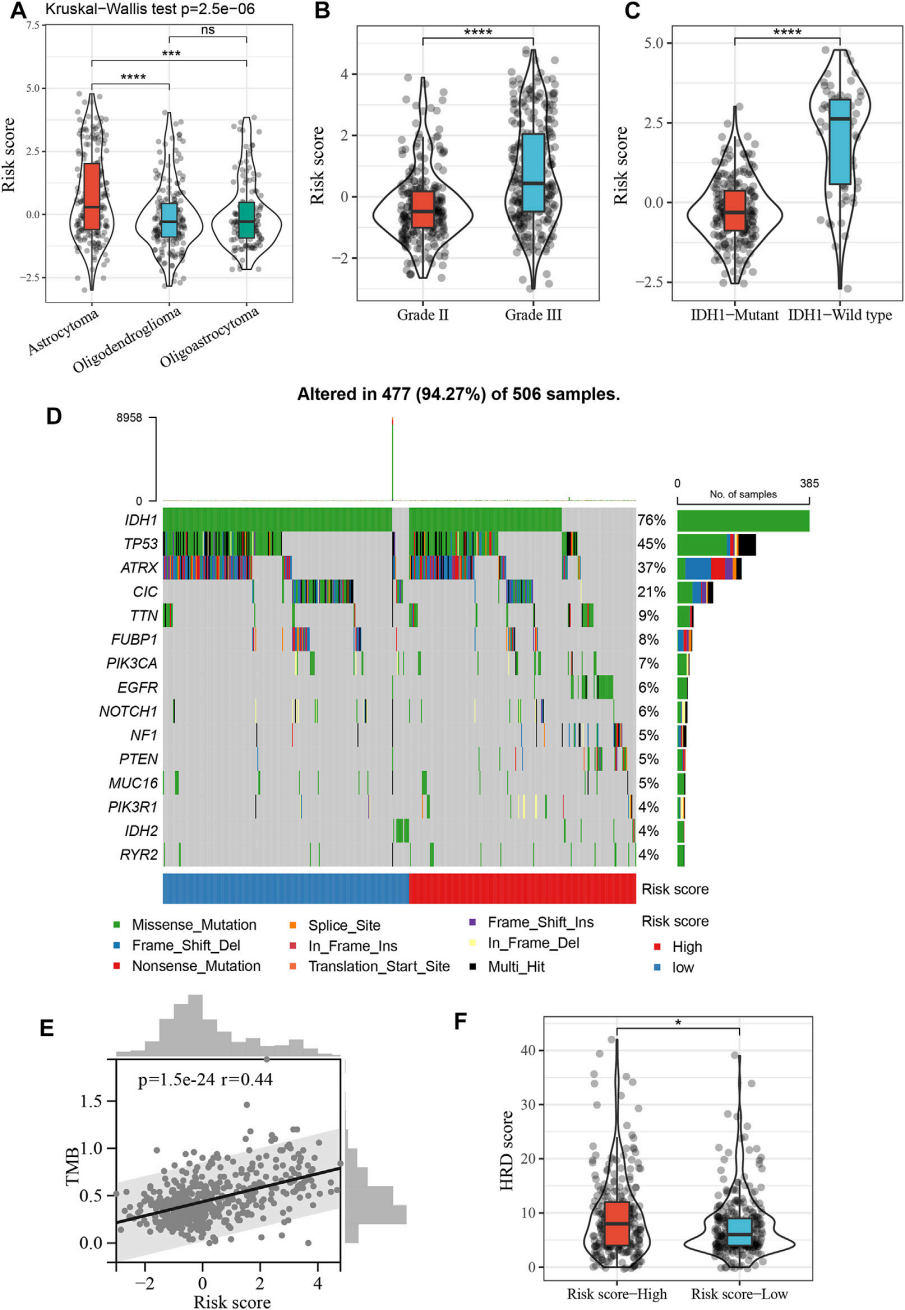
Clinical and mutational characteristics of risk score. **(A)**, Violin plot showing differences in risk score between different histopathological subtypes. ***p < 0.001, ****p < 0.0001. **(B)**, Violin plot showing differences in risk score between grade II and grade III. ****p < 0.0001. **(C)**, Violin plot showing differences in risk score between IDH1 mutant tumor and IDH1 wild-type tumor. ****p < 0.0001. **(D)**, The top 15 frequently mutated genes in high- and low-risk groups. **(E)**, Positive correlation between the TMB and risk score in the TCGA cohort (Spearman’s rank correlation coefficient, *r* = 0.44, *p* = 1.5e−24). **(F)**, Violin plot showing differences in HRD score between high- and low-risk score groups. *p < 0.05.

First, we compared risk scores in LGG for different histological subtypes, and found that the risk score was significantly higher for astrocytomas than for oligodendrogliomas and oligoastrocytomas (*p* < 0.001, Figure 6A). The risk score for grade III tumors was also significantly higher than that for grade II tumors (*p* < 0.0001, Figure 6B). In addition, the risk score for IDH1 wild-type tumors was also higher than that for IDH1 mutant tumors (*p* < 0.0001, Figure 6C). Figure 6D shows the distribution of the 15 most frequently mutated genes in LGG, including IDH1, in the high- and low-risk groups. We also calculated the tumor mutation burden (TMB) for each LGG patient based on the mutation dataset of the TCGA cohort and found a significant positive correlation between TMB and risk score (Figure 6E). Additionally, the high-risk score group had significantly higher HRD scores than the low-risk score group (*p* < 0.05, Figure 6F).

### Molecular Characteristics of Risk Score

**FIGURE 7:**
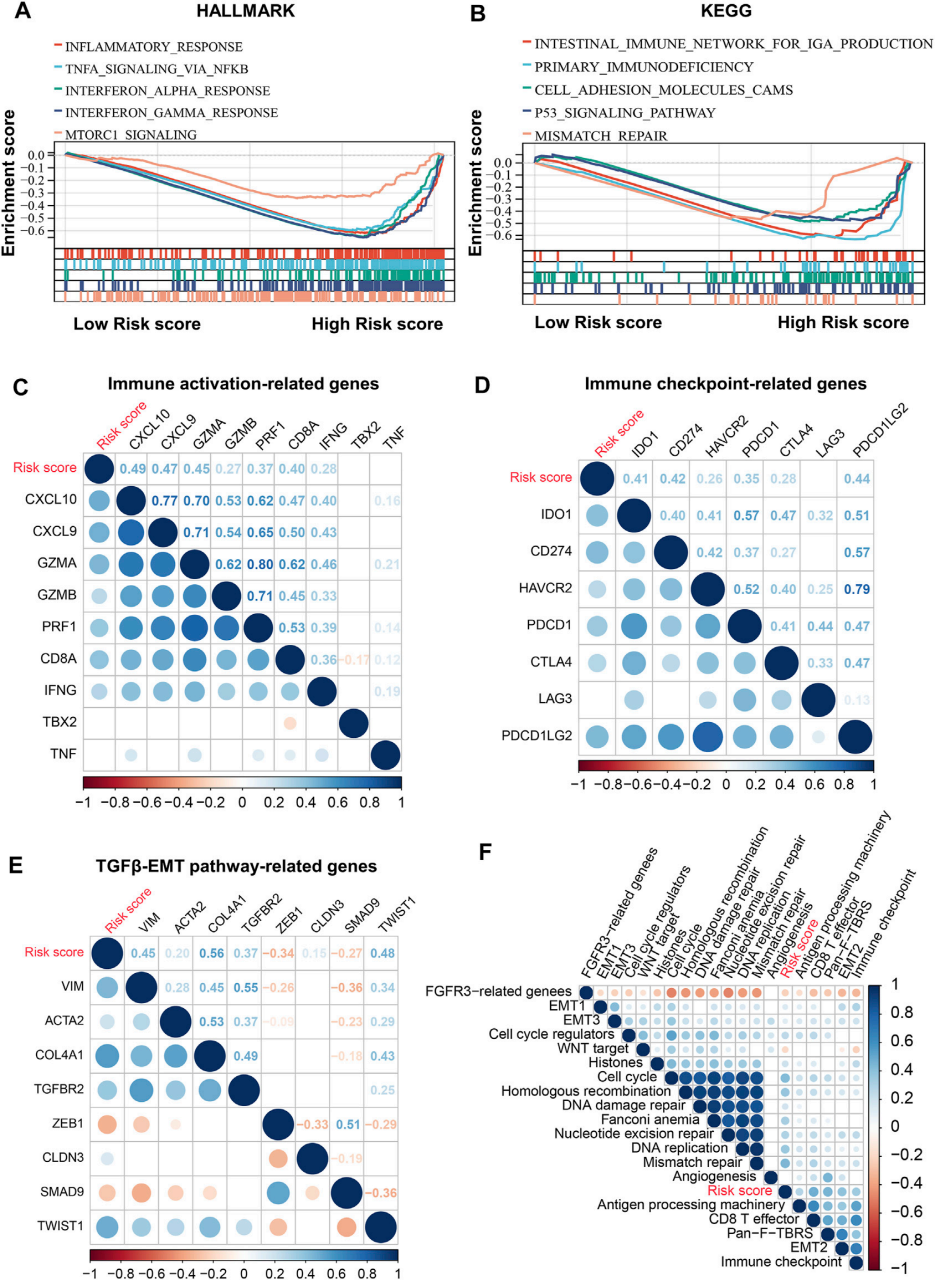
Molecular characteristics of risk score. **(A,B)**, GSEA enrichment plots base on HALLMARK **(A)** and KEGG **(B)** gene sets showing the relatively significantly enriched pathways in high-risk score group. **(C)**, Correlations between risk score and immune activation-related genes expression. **(D)**, Correlations between risk score and immune checkpoint-related genes expression. **(E)**, Correlations between risk score and TGFβ/EMT pathway-related genes expression. **(F)**, Correlations between risk score and core biological pathway signature scores. Correlation coefficients are calculated by Spearman’s correlation analysis, with red representing negative correlations and blue representing positive correlations.

To decipher the potential mechanisms of risk score, we performed GSEA and found that high-risk patients were enriched not only for pro-cancer-related pathways such as mTOR and P53 pathways, but also for immune- and stromal-related processes such as inflammatory response, IFNγ response, TNFα signaling, and cell adhesion (Figures 7A,B). Therefore, we further explored the relationship between the risk scores and immune-related gene sets. The risk score was found to be significantly positively associated not only with most immune activation-related genes, but also with immune checkpoint-related genes, including CTLA4, PDCD1 (PD-1), and CD274 (PD-L1) (Figures 7C,D). In addition, the risk score was also positively associated with the TGFβ/EMT pathway-related genes VIM, ACTA2, COL4A1, TGFBR2, and TWIST1 (Figure 7E). In the core biological pathway analysis, the risk score was positively correlated with most of the genomic and immune signature scores, such as DNA damage repair, DNA replication, cell cycle, homologous recombination, CD8 T-effector, Pan-F-TBRS, immune checkpoint, and EMT (Figure 7F).

### Relationship Between Risk Score and Immune Cell Infiltration

**FIGURE 8:**
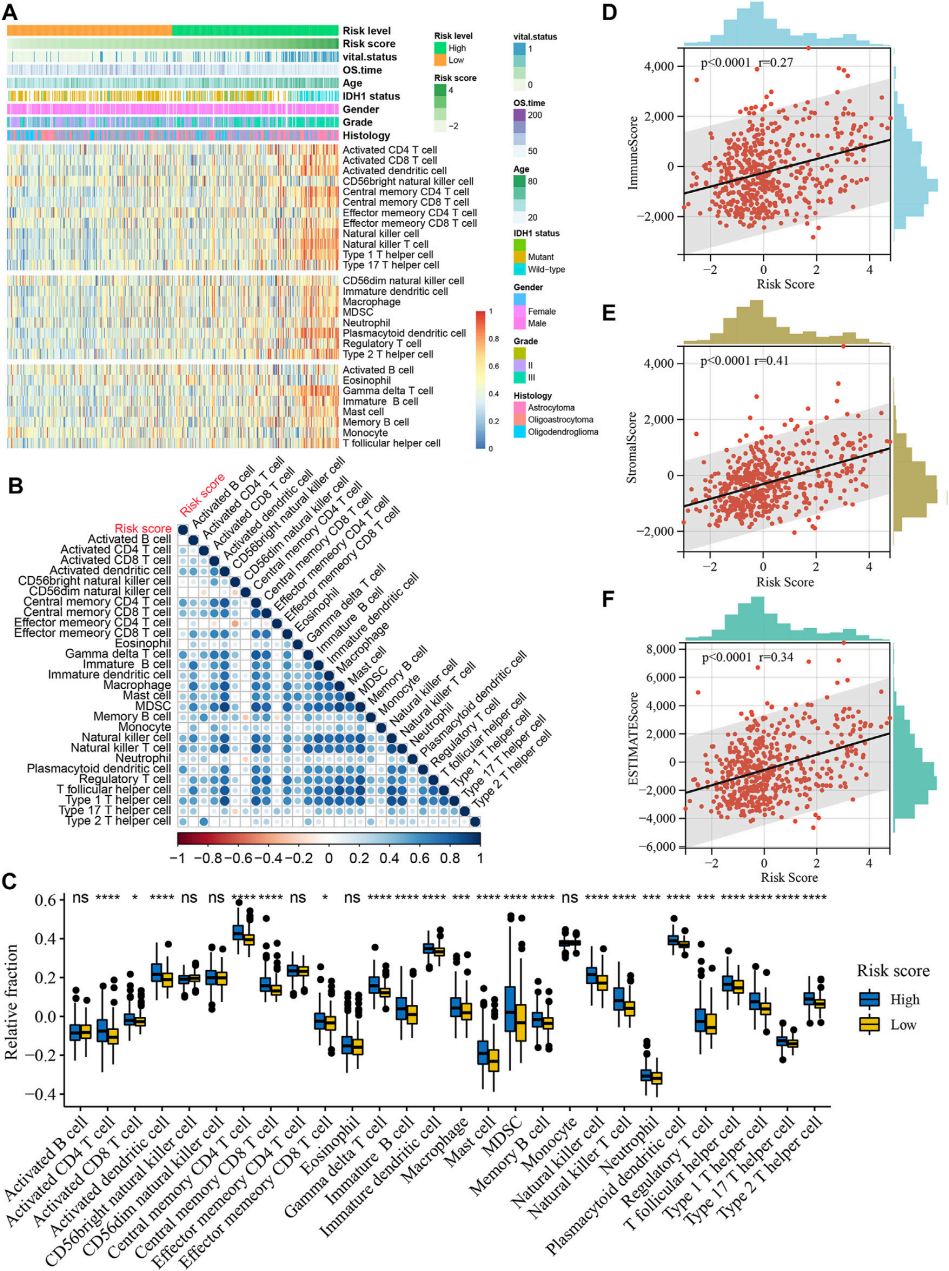
Relationship between risk score and immune cell infiltration in the TCGA cohort. **(A)**, Heatmap of the relationship between risk score and 28 immune cells in the TCGA cohort. Age, IDH1 status, gender, vital status, OS time, histologic subtype and WHO grade are shown as patient annotations. **(B)**, Correlations of risk score with abundance of 28 immune cells. Correlation coefficients are calculated by Spearman’s correlation analysis, with red representing negative correlations and blue representing positive correlations. **(C)**, Boxplots of the relationship between risk score and 28 immune cells. The upper and lower ends of the boxes represented interquartile range of values. The lines in the boxes represented median value, and black dots showed outliers. *p < 0.05, ***p < 0.001, ****p < 0.0001. **(D)**, Positive correlation between the ImmuneScore and risk score (Spearman’s rank correlation coefficient, *r* = 0.27, *p* < 0.0001). **(E)**, Positive correlation between the StromalScore and risk score (Spearman’s rank correlation coefficient, *r* = 0.41, *p* < 0.0001). **(F)**, Positive correlation between the ESTIMATEScore and risk score (Spearman’s rank correlation coefficient, *r* = 0.34, *p* < 0.0001).

To further resolve the relationship between risk score and tumor immune microenvironment (TIME) in LGG, we inferred the infiltration abundance of 28 immune cell species in the TCGA cohort. The distribution of the immune cell infiltrate is illustrated in Figure 8A, and the clinicopathological features of LGG are also included. We found that most immunostimulatory cells (such as activated CD8 T cells and natural killer cells) and immunosuppressive cells (such as macrophages and regulatory T cells) were more abundant in the high-risk group (Figure 8C). In addition, the infiltration level of most immune cells positively correlated with the risk score (Figure 8B). TIME analysis based on the ESTIMATE algorithm showed a significant positive correlation between the risk score and ImmuneScore, StromalScore, and ESTIMATEScore (Figures 8D–F. It is worth noting that these results were validated in the CGGA and Rembrandt cohorts (Supplementary Figures S4, S5).

## Discussion

The association between HRD score and tumor has been uncovered in a variety of tumors, such as in high-grade serous ovarian carcinoma, where the HRD score can classify patients into different prognostic subtypes for personalized treatment (Takaya et al., 2020). However, the role of HRD scores in LGG has been less studied. In this big data study, we determined for the first time that a threshold of HRD score = 10 could classify LGG patients into subgroups with different prognoses, screened potential HRD-related genes, and constructed a robust risk model. In addition, this study correlated HRD with immune response in LGG for the first time, which provides another perspective to further understand the value of HRD in LGG.

In this study, we found that patients with high HRD scores had higher genomic instability. It is well known that genomic instability is critical for tumor development (Negrini et al., 2010; Andor et al., 2017). However, from another perspective, high genomic instability may also result in a higher neoantigen load to tumors, thus making it more likely to be recognized by the immune system and trigger immune responses (Germano et al., 2017; Mardis, 2019). In addition, genomic instability upregulates the cGAS-STING pathway and activates anti-tumor immunity (Chen et al., 2020a; Kwon and Bakhoum, 2020). Consistent with this, GSEA found that LGG patients with high HRD were enriched for inflammatory responses and IFNγ responses, leading to the inference that the higher immune response in patients with high HRD may be due to genomic instability. HRD score was also found to predict the immunogenicity of BRCA1/2 breast cancer in the study by Kraya et al. (2019). Li et al. (2021) found that prostate cancer patients with high HRD had higher immune infiltration and immune checkpoint gene expression, suggesting that prostate cancer patients with high HRD may respond to immune checkpoint inhibitors (ICIs). These studies further supported our results and demonstrated that ICIs may be a promising treatment modality for LGG patients with high HRD. Since monotherapy is often ineffective and PARP inhibitors are more sensitive and may increase chemosensitivity in patients with high HRD (Gupta et al., 2014; Lesueur et al., 2017; Rescigno et al., 2018; Gupta et al., 2019; Chen et al., 2021; Ohba et al., 2021), combining PARP inhibitors with chemotherapy and ICIs may be a potentially effective strategy in patients with high HRD.

In the univariate Cox regression analysis, we found that most of the HRD-related genes (72.5%) were associated with the prognosis of LGG, which implied the key role of HRD-related genes in LGG. Subsequently, combining LASSO-Cox regression and stepwise regression, we constructed a robust risk score model with 11 genes and validated it in an independent cohort. Some of these genes have been reported in previous studies of gliomas. For example, KCNK3 was also identified as a prognosis-related gene in LGG and was associated with the development of LGG in the study by Wu et al. (2022). The downregulation of ASPM can affect DNA double-strand repair by the DNA-PK pathway and enhance the sensitivity of radiotherapy in glioma cells (Kato et al., 2011), while high expression of ASPM is negatively related to TMZ sensitivity in LGG (Wang et al., 2019). In addition, ASPM promotes glioma malignancy by activating the Wnt/β-Catenin signaling pathway (Chen et al., 2020b). CRNDE expression is elevated in gliomas and correlates with glioma grade and histopathological subtype (Ellis et al., 2012). Matsunaga et al. (2021) found that the downregulation of ATP8A2 in C6 glioma cells cultured under serum-free conditions inhibited the stress-induced externalization of annexin A2 and ablated membrane lipid asymmetry. A study by Liu et al. (2018) revealed that SPOCD1 promotes proliferation and metastasis of glioma cells by upregulating the expression of Pentraxin 3. These studies provide support for the prognostic role of risk scores, and our study also provides potential targets for the development of future targeted therapies.

This is the first study to develop a risk model based on HRD in LGG. The HRD-related genes that constitute the risk model are not only potential therapeutic targets, but also demonstrate the critical role of HRD in LGG prognosis, which may inform further HRD-related studies. It is well known that risk stratification is essential for individualized treatment and management of cancer patients. (Watson et al., 2012) Understanding the postoperative risk stratification has a guiding role in early intervention and in the development of individualized treatment plans. (Brana and Siu, 2012; Watson et al., 2012) Currently, in clinical work, prognosis is usually predicted by clinical parameters of LGG patients, such as tumor grade, histological classification, and IDH1 status. (Zhang et al., 2013; Komori et al., 2018; McFaline-Figueroa and Lee, 2018; Kwon et al., 2020). In the present study we propose a more accurate risk stratification model. By comparing the C-index between the risk model and other clinical parameters, we found that the risk model had the highest C-index in both the TCGA and CGGA cohorts (Supplementary Table S6). Notably, although the risk model was constructed based on RNA-seq data, our results also confirm its availability in microarray data (Rembrandt cohort). Furthermore, according to the coefficients of HRD-related genes in the risk model, higher expression of genes with positive correlation coefficients is associated with higher risk, while the opposite is true for genes with negative correlation coefficients. Therefore, it is possible to roughly infer risk scores based only on the expression levels of some of the genes in the risk model, which was also confirmed in the Rembrandt cohort. We provide an R script to facilitate the inference of risk scores and immune characteristics for individual samples (Supplementary File S1).

In the present study, we demonstrated that patients with high-risk scores had higher immune cell infiltration and higher immune responses, as well as higher stromal activation and infiltration of immunosuppressive cells, implying that high-risk patients had “hot” and suppressed TIME. Although previous studies have demonstrated that pre-existing antitumor immunity is beneficial to tumor patient survival (Rooney et al., 2015; Li et al., 2019a), the prognosis of high-risk patients who exhibited “hot” TIME in this study had significantly worse survival, possibly due to intense immunosuppression. Consistent with this, previous studies have also shown that immunosuppression has a critical impact on the prognosis of patients with glioma (Sampson et al., 2017; Tomaszewski et al., 2019). Given that high-risk patients also have higher TMB and immune checkpoint molecule expression, it is reasonable to assume that treatment with ICIs in high-risk patients could attenuate immunosuppression to enhance existing antitumor immunity. In contrast, low-risk patients with “cold” TIME may be suitable for immunostimulatory agents such as tumor vaccines to increase anti-tumor immune cell infiltration. However, ICI monotherapy is not currently effective enough in gliomas (Sampson et al., 2015; Li et al., 2019b), and new strategies are using combination therapies such as ICIs combined with tumor vaccines to stimulate immune responses against the tumor (Vázquez Cervantes et al., 2021). Notably, high-risk patients also had higher HRD scores, which makes the strategy of adding PARP inhibitors to combination therapy worth trying.

This study has several limitations. First, we were unable to collect LGG samples treated with PARP inhibitors and ICIs to confirm the speculation that patients with high HRD scores or high-risk scores may benefit from PARP inhibitors and ICIs. Second, the prognostic, predictive role of risk score needs to be validated in prospective cohorts, yet this may require decades of follow-up. In addition, the biological role of genes in the risk model should be elucidated in further preclinical studies in LGG.

## Conclusion

In conclusion, this study determined the threshold for HRD score in LGG for the first time, identified HRD-related genes, and constructed an HRD-based risk score model. Patients with high HRD scores and high risk scores may benefit from PARP inhibitors and ICIs. The risk score may not only serve as an effective prognostic marker, but may also provide potential new targets for future targeted therapies and facilitate the development of individualized treatment strategies.

## Data Availability

The datasets presented in this study can be found in online repositories. The names of the repository/repositories and accession number(s) can be found in the article/Supplementary Material.

## Abbreviations

AUC, Area under the curve; CGGA, Chinese Glioma Genome Atlas; DEG, Differentially expressed gene; EMT, Epithelial-mesenchymal transition; FC, Fold change; GO, Gene Ontology; GSEA, Gene set enrichment analysis; HRD, Homologous recombination deficiency; IDH1, Isocitrate dehydrogenase 1; IFNγ, Interferon-gamma; KEGG, Kyoto Encyclopedia of Genes and Genomes; KM, Kaplan-Meier; LASSO, Least absolute shrinkage and selection operator; LGG, Lower grade glioma; LOH, Loss of heterozygosity; LST, Large-scale state transition; MGMT, O6-methylguanine DNA methyltransferase; MSI, Microsatellite instability; OS, Overall survival; Pan-F-TBRS, Pan-fibroblast TGFβ response signature; PARP, Poly ADP-ribose polymerase; ROC, Receiver operating characteristic; ssGSEA, Single-sample gene set enrichment analysis; TAI, Telomeric allelic imbalance; TCGA, The Cancer Genome Atlas; TGF, Transforming growth factor; TIME, Tumor immune microenvironment; TMZ, Temozolomide; WHO, World Health Organization.

## References

[B1] Abkevich V. Timms K. M. Hennessy B. T. Potter J. Carey M. S. Meyer L. A. (2012). Patterns of Genomic Loss of Heterozygosity Predict Homologous Recombination Repair Defects in Epithelial Ovarian Cancer. Br. J. Cancer 107 (10), 1776–1782. 10.1038/bjc.2012.451 PubMed Abstract | 10.1038/bjc.2012.451 | Google Scholar 23047548 PMC3493866

[B2] Andor N. Maley C. C. Ji H. P. (2017). Genomic Instability in Cancer: Teetering on the Limit of Tolerance. Cancer Res. 77 (9), 2179–2185. 10.1158/0008-5472.CAN-16-1553 PubMed Abstract | 10.1158/0008-5472.CAN-16-1553 | Google Scholar 28432052 PMC5413432

[B3] Appin C. L. Brat D. J. (2014). Molecular Genetics of Gliomas. Cancer J. (United States) 20 (1), 66–72. 10.1097/PPO.0000000000000020 PubMed Abstract | 10.1097/PPO.0000000000000020 | Google Scholar 24445767

[B4] Binabaj M. M. Bahrami A. ShahidSales S. Joodi M. Joudi Mashhad M. Hassanian S. M. (2018). The Prognostic Value of MGMT Promoter Methylation in Glioblastoma: A Meta‐analysis of Clinical Trials. J. Cell. Physiol. 233 (1), 378–386. 10.1002/jcp.25896 PubMed Abstract | 10.1002/jcp.25896 | Google Scholar 28266716

[B5] Birkbak N. J. Wang Z. C. Kim J.-Y. Eklund A. C. Li Q. Tian R. (2012). Telomeric Allelic Imbalance Indicates Defective DNA Repair and Sensitivity to DNA-Damaging Agents. Cancer Discov. 2 (4), 366–375. 10.1158/2159-8290.CD-11-0206 PubMed Abstract | 10.1158/2159-8290.CD-11-0206 | Google Scholar 22576213 PMC3806629

[B6] Bowman R. L. Wang Q. Carro A. Verhaak R. G. W. Squatrito M. (2017). GlioVis Data Portal for Visualization and Analysis of Brain Tumor Expression Datasets. Neuonc 19 (1), 139–141. 10.1093/neuonc/now247 PubMed Abstract | 10.1093/neuonc/now247 | Google Scholar PMC5193031 28031383

[B7] Brana I. Siu L. L. (2012). Locally Advanced Head and Neck Squamous Cell Cancer: Treatment Choice Based on Risk Factors and Optimizing Drug Prescription. Ann. Oncol. 23 (Suppl. 10), x178–x185. 10.1093/annonc/mds322 PubMed Abstract | 10.1093/annonc/mds322 | Google Scholar 22987958

[B8] Charoentong P. Finotello F. Angelova M. Mayer C. Efremova M. Rieder D. (2017). Pan-cancer Immunogenomic Analyses Reveal Genotype-Immunophenotype Relationships and Predictors of Response to Checkpoint Blockade. Cell. Rep. 18 (1), 248–262. 10.1016/j.celrep.2016.12.019 PubMed Abstract | 10.1016/j.celrep.2016.12.019 | Google Scholar 28052254

[B9] Chen C. Cheng X. Li S. Chen H. Cui M. Bian L. (2021). A Novel Signature for Predicting Prognosis of Smoking-Related Squamous Cell Carcinoma. Front. Genet. 12. 10.3389/fgene.2021.666371 10.3389/fgene.2021.666371 | Google Scholar PMC8100348 33968141

[B10] Chen H. Chen H. Zhang J. Wang Y. Simoneau A. Yang H. (2020). cGAS Suppresses Genomic Instability as a Decelerator of Replication Forks. Sci. Adv. 6 (42). 10.1126/sciadv.abb8941 PubMed Abstract | 10.1126/sciadv.abb8941 | Google Scholar PMC7556829 33055160

[B11] Chen X. Huang L. Yang Y. Chen S. Sun J. Ma C. (2020). ASPM Promotes Glioblastoma Growth by Regulating G1 Restriction Point Progression and Wnt-β-Catenin Signaling. Aging 12 (1), 224–241. 10.18632/aging.102612 PubMed Abstract | 10.18632/aging.102612 | Google Scholar 31905171 PMC6977704

[B12] Comprehensive (2015). Comprehensive, Integrative Genomic Analysis of Diffuse Lower-Grade Gliomas. N. Engl. J. Med. 372 (26), 2481–2498. 10.1056/nejmoa1402121 PubMed Abstract | 10.1056/nejmoa1402121 | Google Scholar 26061751 PMC4530011

[B13] Davies H. Glodzik D. Morganella S. Yates L. R. Staaf J. Zou X. (2017). HRDetect Is a Predictor of BRCA1 and BRCA2 Deficiency Based on Mutational Signatures. Nat. Med. 23 (4), 517–525. 10.1038/nm.4292 PubMed Abstract | 10.1038/nm.4292 | Google Scholar 28288110 PMC5833945

[B14] Eckel-Passow J. E. Lachance D. H. Molinaro A. M. Walsh K. M. Decker P. A. Sicotte H. (2015). Glioma Groups Based on 1p/19q,IDH, andTERTPromoter Mutations in Tumors. N. Engl. J. Med. 372 (26), 2499–2508. 10.1056/nejmoa1407279 PubMed Abstract | 10.1056/nejmoa1407279 | Google Scholar 26061753 PMC4489704

[B15] Ellis B. C. Molloy P. L. Graham L. D. (2012). CRNDE: A Long Non-coding RNA Involved in CanceR, Neurobiology, and DEvelopment. Front. Gene. 3 (NOV), 1–15. 10.3389/FGENE.2012.00270 PubMed Abstract | 10.3389/FGENE.2012.00270 | Google Scholar PMC3509318 23226159

[B16] Germano G. Lamba S. Rospo G. Barault L. Magrì A. Maione F. (2017). Inactivation of DNA Repair Triggers Neoantigen Generation and Impairs Tumour Growth. Nature 552 (7683), 116–120. 10.1038/nature24673 PubMed Abstract | 10.1038/nature24673 | Google Scholar 29186113

[B17] Gupta S. K. Mladek A. C. Carlson B. L. Boakye-Agyeman F. Bakken K. K. Kizilbash S. H. (2014). Discordant *In Vitro* and *In Vivo* Chemopotentiating Effects of the PARP Inhibitor Veliparib in Temozolomide-Sensitive versus -resistant Glioblastoma Multiforme Xenografts. Clin. Cancer Res. 20 (14), 3730–3741. 10.1158/1078-0432.CCR-13-3446 PubMed Abstract | 10.1158/1078-0432.CCR-13-3446 | Google Scholar 24838527 PMC4111895

[B18] Gupta S. K. Smith E. J. Mladek A. C. Tian S. Decker P. A. Kizilbash S. H. (2019). PARP Inhibitors for Sensitization of Alkylation Chemotherapy in Glioblastoma: Impact of Blood-Brain Barrier and Molecular Heterogeneity. Front. Oncol. 8 (JAN). 10.3389/fonc.2018.00670 PubMed Abstract | 10.3389/fonc.2018.00670 | Google Scholar PMC6349736 30723695

[B19] Jiang T. Mao Y. Ma W. Mao Q. You Y. Yang X. (2016). CGCG Clinical Practice Guidelines for the Management of Adult Diffuse Gliomas. Cancer Lett. 375 (2), 263–273. 10.1016/j.canlet.2016.01.024 PubMed Abstract | 10.1016/j.canlet.2016.01.024 | Google Scholar 26966000

[B20] Jonsson P. Bandlamudi C. Cheng M. L. Srinivasan P. Chavan S. S. Friedman N. D. (2019). Tumour Lineage Shapes BRCA-Mediated Phenotypes. Nature 571 (7766), 576–579. 10.1038/S41586-019-1382-1 PubMed Abstract | 10.1038/S41586-019-1382-1 | Google Scholar 31292550 PMC7048239

[B21] Kato T. A. Okayasu R. Jeggo P. A. Fujimori A. (2011). ASPM Influences DNA Double-Strand Break Repair and Represents a Potential Target for Radiotherapy. Int. J. Radiat. Biol. 87 (12), 1189–1195. 10.3109/09553002.2011.624152 PubMed Abstract | 10.3109/09553002.2011.624152 | Google Scholar 21923303

[B22] Komori T. Muragaki Y. Chernov M. F. (2018). Pathology and Genetics of Gliomas. Prog. Neurol. Surg. 31, 1–37. 10.1159/000466835 PubMed Abstract | 10.1159/000466835 | Google Scholar 29393190

[B23] Kraya A. A. Maxwell K. N. Wubbenhorst B. Wenz B. M. Pluta J. Rech A. J. (2019). Genomic Signatures Predict the Immunogenicity of BRCA-Deficient Breast Cancer. Clin. Cancer Res. 25 (14), 4363–4374. 10.1158/1078-0432.CCR-18-0468 PubMed Abstract | 10.1158/1078-0432.CCR-18-0468 | Google Scholar 30914433 PMC6635013

[B24] Kwon J. Bakhoum S. F. (2020). The Cytosolic DNA-Sensing cGAS-STING Pathway in Cancer. Cancer Discov. 10 (1), 26–39. 10.1158/2159-8290.CD-19-0761 PubMed Abstract | 10.1158/2159-8290.CD-19-0761 | Google Scholar 31852718 PMC7151642

[B25] Kwon M. J. Kang S. Y. Cho H. Lee J. I. Kim S. T. Suh Y. L. (2020). Clinical Relevance of Molecular Subgrouping of Gliomatosis Cerebri Per 2016 WHO Classification: a Clinicopathological Study of 89 Cases. Brain Pathol. 30 (2), 235–245. 10.1111/bpa.12782 PubMed Abstract | 10.1111/bpa.12782 | Google Scholar 31435963 PMC8018049

[B26] Ladan M. M. van Gent D. C. Jager A. (2021). Homologous Recombination Deficiency Testing for Brca-like Tumors: The Road to Clinical Validation. Cancers 13 (5), 1004–1023. 10.3390/cancers13051004 PubMed Abstract | 10.3390/cancers13051004 | Google Scholar 33670893 PMC7957671

[B27] Lapointe S. Perry A. Butowski N. A. (2018). Primary Brain Tumours in Adults. Lancet 392 (10145), 432–446. 10.1016/S0140-6736(18)30990-5 PubMed Abstract | 10.1016/S0140-6736(18)30990-5 | Google Scholar 30060998

[B28] Lesueur P. Chevalier F. Austry J.-B. Waissi W. Burckel H. Noël G. (2017). Poly-(ADP-ribose)-polymerase Inhibitors as Radiosensitizers: A Systematic Review of Pre-clinical and Clinical Human Studies. Oncotarget 8 (40), 69105–69124. 10.18632/oncotarget.19079 PubMed Abstract | 10.18632/oncotarget.19079 | Google Scholar 28978184 PMC5620324

[B29] Li B. Chan H. L. Chen P. (2019). Immune Checkpoint Inhibitors: Basics and Challenges. Cmc 26 (17), 3009–3025. 10.2174/0929867324666170804143706 PubMed Abstract | 10.2174/0929867324666170804143706 | Google Scholar 28782469

[B30] Li B. Cui Y. Nambiar D. K. Sunwoo J. B. Li R. (2019). The Immune Subtypes and Landscape of Squamous Cell Carcinoma. Clin. Cancer Res. 25 (12), clincanres.4085.2018–3537. 10.1158/1078-0432.CCR-18-4085 PubMed Abstract | 10.1158/1078-0432.CCR-18-4085 | Google Scholar PMC6571041 30833271

[B31] Li Y. Zhao Z. Ai L. Wang Y. Liu K. Chen B. (2021). Discovering a Qualitative Transcriptional Signature of Homologous Recombination Defectiveness for Prostate Cancer. iScience 24 (10), 103135. 10.1016/J.ISCI.2021.103135 PubMed Abstract | 10.1016/J.ISCI.2021.103135 | Google Scholar 34622176 PMC8482486

[B32] Liu Q. Wang X. Y. Qin Y. Y. Yan X. L. Chen H. M. Huang Q. D. (2018). SPOCD1 Promotes the Proliferation and Metastasis of Glioma Cells by Up-Regulating PTX3. Am. J. Cancer Res. 8 (4), 624–635. PubMed Abstract | Google Scholar 29736308 PMC5934553

[B33] Manié E. Popova T. Battistella A. Tarabeux J. Caux-Moncoutier V. Golmard L. (2016). Genomic Hallmarks of Homologous Recombination Deficiency in Invasive Breast Carcinomas. Int. J. Cancer 138 (4), 891–900. 10.1002/ijc.29829 PubMed Abstract | 10.1002/ijc.29829 | Google Scholar 26317927

[B34] Mardis E. R. (2019). Neoantigens and Genome Instability: Impact on Immunogenomic Phenotypes and Immunotherapy Response. Genome Med. 11 (1). 10.1186/s13073-019-0684-0 PubMed Abstract | 10.1186/s13073-019-0684-0 | Google Scholar PMC6865009 31747945

[B35] Mariathasan S. Turley S. J. Nickles D. Castiglioni A. Yuen K. Wang Y. (2018). TGFβ Attenuates Tumour Response to PD-L1 Blockade by Contributing to Exclusion of T Cells. Nature 554 (7693), 544–548. 10.1038/nature25501 PubMed Abstract | 10.1038/nature25501 | Google Scholar 29443960 PMC6028240

[B36] Matsunaga H. Halder S. K. Ueda H. (2021). Annexin A2 Flop-Out Mediates the Non-vesicular Release of DAMPs/Alarmins from C6 Glioma Cells Induced by Serum-free Conditions. Cells 10 (3), 567. 10.3390/cells10030567 PubMed Abstract | 10.3390/cells10030567 | Google Scholar 33807671 PMC7998613

[B37] McFaline-Figueroa J. R. Lee E. Q. (2018). Brain Tumors. Am. J. Med. 131 (8), 874–882. 10.1016/j.amjmed.2017.12.039 PubMed Abstract | 10.1016/j.amjmed.2017.12.039 | Google Scholar 29371158

[B38] Negrini S. Gorgoulis V. G. Halazonetis T. D. (2010). Genomic Instability - an Evolving Hallmark of Cancer. Nat. Rev. Mol. Cell. Biol. 11 (3), 220–228. 10.1038/nrm2858 PubMed Abstract | 10.1038/nrm2858 | Google Scholar 20177397

[B39] Ohba S. Yamashiro K. Hirose Y. (2021). Inhibition of Dna Repair in Combination with Temozolomide or Dianhydrogalactiol Overcomes Temozolomide-Resistant Glioma Cells. Cancers 13 (11), 2570. 10.3390/cancers13112570 PubMed Abstract | 10.3390/cancers13112570 | Google Scholar 34073837 PMC8197190

[B40] Peng G. Chun-Jen Lin C. Mo W. Dai H. Park Y.-Y. Kim S. M. (2014). Genome-wide Transcriptome Profiling of Homologous Recombination DNA Repair. Nat. Commun. 5, 3361. 10.1038/ncomms4361 PubMed Abstract | 10.1038/ncomms4361 | Google Scholar 24553445 PMC4017859

[B41] Rescigno P. Chandler R. de Bono J. (2018). Relevance of Poly (ADP-Ribose) Polymerase Inhibitors in Prostate Cancer. Curr. Opin. Support Palliat. Care 12 (3), 339–343. 10.1097/SPC.0000000000000358 PubMed Abstract | 10.1097/SPC.0000000000000358 | Google Scholar 29979319

[B42] Rooney M. S. Shukla S. A. Wu C. J. Getz G. Hacohen N. (2015). Molecular and Genetic Properties of Tumors Associated with Local Immune Cytolytic Activity. Cell. 160 (1-2), 48–61. 10.1016/j.cell.2014.12.033 PubMed Abstract | 10.1016/j.cell.2014.12.033 | Google Scholar 25594174 PMC4856474

[B43] Sampson J. H. Maus M. V. June C. H. (2017). Immunotherapy for Brain Tumors. Jco 35 (21), 2450–2456. 10.1200/JCO.2017.72.8089 PubMed Abstract | 10.1200/JCO.2017.72.8089 | Google Scholar 28640704

[B44] Sampson J. H. Vlahovic G. Sahebjam S. Omuro A. M. P. Baehring J. M. Hafler D. A. (2015). Preliminary Safety and Activity of Nivolumab and its Combination with Ipilimumab in Recurrent Glioblastoma (GBM): CHECKMATE-143. Jco 33 (15_Suppl. l), 3010. 10.1200/jco.2015.33.15_suppl.3010 10.1200/jco.2015.33.15_suppl.3010 | Google Scholar

[B45] Subramanian A. Tamayo P. Mootha V. K. Mukherjee S. Ebert B. L. Gillette M. A. (2005). Gene Set Enrichment Analysis: A Knowledge-Based Approach for Interpreting Genome-wide Expression Profiles. Proc. Natl. Acad. Sci. U.S.A. 102 (43), 15545–15550. 10.1073/pnas.0506580102 PubMed Abstract | 10.1073/pnas.0506580102 | Google Scholar 16199517 PMC1239896

[B46] Sztupinszki Z. Diossy M. Krzystanek M. Borcsok J. Pomerantz M. M. Tisza V. (2020). Detection of Molecular Signatures of Homologous Recombination Deficiency in Prostate Cancer with or without BRCA1/2 Mutations. Clin. Cancer Res. 26 (11), 2673–2680. 10.1158/1078-0432.CCR-19-2135 PubMed Abstract | 10.1158/1078-0432.CCR-19-2135 | Google Scholar 32071115 PMC8387086

[B47] Sztupinszki Z. Diossy M. Krzystanek M. Reiniger L. Csabai I. Favero F. (2018). Migrating the SNP Array-Based Homologous Recombination Deficiency Measures to Next Generation Sequencing Data of Breast Cancer. npj Breast Cancer 4 (1), 16. 10.1038/s41523-018-0066-6 PubMed Abstract | 10.1038/s41523-018-0066-6 | Google Scholar 29978035 PMC6028448

[B48] Takaya H. Nakai H. Takamatsu S. Mandai M. Matsumura N. (2020). Homologous Recombination Deficiency Status-Based Classification of High-Grade Serous Ovarian Carcinoma. Sci. Rep. 10 (1). 10.1038/s41598-020-59671-3 10.1038/s41598-020-59671-3 | Google Scholar PMC7026096 32066851

[B49] Telli M. L. Timms K. M. Reid J. Hennessy B. Mills G. B. Jensen K. C. (2016). Homologous Recombination Deficiency (Hrd) Score Predicts Response to Platinum-Containing Neoadjuvant Chemotherapy in Patients with Triple-Negative Breast Cancer. Clin. Cancer Res. 22 (15), 3764–3773. 10.1158/1078-0432.CCR-15-2477 PubMed Abstract | 10.1158/1078-0432.CCR-15-2477 | Google Scholar 26957554 PMC6773427

[B50] Theeler B. J. Yung W. K. A. Fuller G. N. De Groot J. F. (2012). Moving toward Molecular Classification of Diffuse Gliomas in Adults. Neurology 79 (18), 1917–1926. 10.1212/WNL.0b013e318271f7cb PubMed Abstract | 10.1212/WNL.0b013e318271f7cb | Google Scholar 23109653 PMC3525311

[B51] Thorsson V. Gibbs D. L. Brown S. D. Wolf D. Bortone D. S. Ou Yang T. H. (2018). The Immune Landscape of Cancer. Immunity 48 (4), 812–e14. e14. 10.1016/j.immuni.2018.03.023 PubMed Abstract | 10.1016/j.immuni.2018.03.023 | Google Scholar 29628290 PMC5982584

[B52] Tibshirani R. (1997). The Lasso Method for Variable Selection in the Cox Model. Stat. Med. 16 (4), 385–395. 10.1002/(sici)1097-0258(19970228)16:4<385::aid-sim380>3.0.co;2-3 PubMed Abstract | 10.1002/(sici)1097-0258(19970228)16:4<385::aid-sim380>3.0.co;2-3 | Google Scholar 9044528

[B53] Tomaszewski W. Sanchez-Perez L. Gajewski T. F. Sampson J. H. (2019). Brain Tumor Microenvironment and Host State: Implications for Immunotherapy. Clin. Cancer Res. 25 (14), 4202–4210. 10.1158/1078-0432.CCR-18-1627 PubMed Abstract | 10.1158/1078-0432.CCR-18-1627 | Google Scholar 30804019 PMC6635001

[B54] Van Den Bent M. J. (2010). Interobserver Variation of the Histopathological Diagnosis in Clinical Trials on Glioma: a Clinician's Perspective. Acta Neuropathol. 120 (3), 297–304. 10.1007/s00401-010-0725-7 PubMed Abstract | 10.1007/s00401-010-0725-7 | Google Scholar 20644945 PMC2910894

[B55] Vázquez Cervantes G. I. González Esquivel D. F. Gómez-Manzo S. Pineda B. Pérez de la Cruz V. (2021). New Immunotherapeutic Approaches for Glioblastoma. J. Immunol. Res. 2021, 1–19. 10.1155/2021/3412906 10.1155/2021/3412906 | Google Scholar PMC8455182 34557553

[B56] Wang Q. He Z. Chen Y. (2019). Comprehensive Analysis Reveals a 4-Gene Signature in Predicting Response to Temozolomide in Low-Grade Glioma Patients. Cancer control. 26 (1), 107327481985511. 10.1177/1073274819855118 PubMed Abstract | 10.1177/1073274819855118 | Google Scholar PMC6558750 31167546

[B57] Watson E. K. Rose P. W. Neal R. D. Hulbert-Williams N. Donnelly P. Hubbard G. (2012). Personalised Cancer Follow-Up: Risk Stratification, Needs Assessment or Both? Br. J. Cancer 106 (1), 1–5. 10.1038/bjc.2011.535 PubMed Abstract | 10.1038/bjc.2011.535 | Google Scholar 22215103 PMC3251871

[B58] Wu F. Wang Z. L. Wang K. Y. Li G. Z. Chai R. C. Liu Y. Q. (2020). Classification of Diffuse Lower‐grade Glioma Based on Immunological Profiling. Mol. Oncol. 14 (9), 2081–2095. 10.1002/1878-0261.12707 PubMed Abstract | 10.1002/1878-0261.12707 | Google Scholar 32392361 PMC7463381

[B59] Wu Y. M. Sa Y. Guo Y. Li Q. F. Zhang N. (2022). Identification of WHO II/III Gliomas by 16 Prognostic-Related Gene Signatures Using Machine Learning Methods. Cmc 29, 1622–1639. 10.2174/0929867328666210827103049 10.2174/0929867328666210827103049 | Google Scholar 34455959

[B60] Xiao K. Liu Q. Peng G. Su J. Qin C.-Y. Wang X.-Y. (2020). Identification and Validation of a Three-Gene Signature as a Candidate Prognostic Biomarker for Lower Grade Glioma. PeerJ 8 (1), e8312. 10.7717/peerj.8312 PubMed Abstract | 10.7717/peerj.8312 | Google Scholar 31921517 PMC6944128

[B61] Yoshihara K. Shahmoradgoli M. Martínez E. Vegesna R. Kim H. Torres-Garcia W. (2013). Inferring Tumour Purity and Stromal and Immune Cell Admixture from Expression Data. Nat. Commun. 4. 10.1038/ncomms3612 10.1038/ncomms3612 | Google Scholar PMC3826632 24113773

[B62] Yu G. Wang L.-G. Han Y. He Q.-Y. (2012). ClusterProfiler: An R Package for Comparing Biological Themes Among Gene Clusters. OMICS A J. Integr. Biol. 16 (5), 284–287. 10.1089/omi.2011.0118 PubMed Abstract | 10.1089/omi.2011.0118 | Google Scholar PMC3339379 22455463

[B63] Zeng D. Li M. Zhou R. Zhang J. Sun H. Shi M. (2019). Tumor Microenvironment Characterization in Gastric Cancer Identifies Prognostic and Immunotherapeutically Relevant Gene Signatures. Cancer Immunol. Res. 7 (5), 737–750. 10.1158/2326-6066.CIR-18-0436 PubMed Abstract | 10.1158/2326-6066.CIR-18-0436 | Google Scholar 30842092

[B64] Zhang C. Bao Z. Zhang W. Jiang T. (2013). Progress on Molecular Biomarkers and Classification of Malignant Gliomas. Front. Med. 7 (2), 150–156. 10.1007/s11684-013-0267-1 PubMed Abstract | 10.1007/s11684-013-0267-1 | Google Scholar 23681890

[B65] Zhuang S. Chen T. Li Y. Wang Y. Ai L. Geng Y. (2021). A Transcriptional Signature Detects Homologous Recombination Deficiency in Pancreatic Cancer at the Individual Level. Mol. Ther. - Nucleic Acids 26, 1014–1026. 10.1016/J.OMTN.2021.10.014 PubMed Abstract | 10.1016/J.OMTN.2021.10.014 | Google Scholar 34786207 PMC8571416

