# Supplementary material for: A Risk Model Developed Based on Homologous Recombination Deficiency Predicts Overall Survival in Patients With Lower Grade Glioma
Source: Front Genet. 2022 Jul 1;13:919391. doi: 10.3389/fgene.2022.919391 (PMC9283922; doi:10.3389/fgene.2022.919391)
Supplement: Supplementary file 2 [file DataSheet2.docx]

Table S1. Clinical characteristics of LGG patients in this study.

|  | **TCGA cohort (N=506)** | **CGGA cohort (N=431)** |
| --- | --- | --- |
| **Age (%)** |  |  |
| ≤40 years | 249 (49) | 220 (51) |
| >40 years | 257 (51) | 211 (49) |
| **Gender (%)** |  |  |
| Female | 201 (40) | 189 (44) |
| Male | 265 (52) | 242 (56) |
| NA | 40 (8) | - |
| **Grade (%)** |  |  |
| II | 224 (44) | 180 (42) |
| III | 242 (48) | 251 (58) |
| NA | 40 (8) | - |
| **Histology (%)** |  |  |
| Astrocytoma | 193 (38) | 263 (61) |
| Oligoastrocytoma | 129 (25) | 29 (7) |
| Oligodendroglioma | 184 (37) | 139 (32) |
| **Vital status (%)** |  |  |
| Alive | 385 (76) | 242 (56) |
| Dead | 121 (24) | 189 (44) |
| **IDH1 status (%)** |  |  |
| Mutant | 216 (43) | 297 (69) |
| Wild-type | 66 (13) | 96 (22) |
| NA | 224 (44) | 38 (9) |

-, data unavailable; NA, no data.

Table S5. Univariate cox regression analysis to identify risk factors in LGG.

| **Characteristics** | TCGA cohort | | | CGGA cohort | | |
| --- | --- | --- | --- | --- | --- | --- |
|  | HR | 95%CI | *P*-value | HR | 95%CI | *P*-value |
| **Age** (Continuous) | 1.058 | 1.043~1.073 | <0.001 | 1.008 | 0.993~1.022 | 0.275 |
| **Histology** |  |  |  |  |  |  |
| Astrocytoma | (Reference) | | | (Reference) | | |
| Oligoastrocytoma | 0.602 | 0.375~0.967 | 0.035 | 0.577 | 0.312~1.067 | 0.079 |
| Oligodendroglioma | 0.578 | 0.384~0.870 | 0.008 | 0.333 | 0.230~0.483 | <0.001 |
| **Grade** (III vs. II) | 2.875 | 1.886~4.382 | <0.001 | 2.621 | 1.887~3.642 | <0.001 |
| **Gender** (Male vs. Female) | 0.975 | 0.660~1.441 | 0.902 | 0.999 | 0.749~1.333 | 0.997 |
| **IDH1 status** (Wild-type vs. Mutant) | 4.582 | 2.799~7.502 | <0.001 | 2.246 | 1.643~3.070 | <0.001 |
| **Risk score** | 2.718 | 2.350~3.144 | <0.001 | 2.613 | 2.143~3.187 | <0.001 |

Table S6. C-index of clinical characteristics in this study.

|  | **TCGA cohort** | **CGGA cohort** |
| --- | --- | --- |
| **Age** | 0.751 | 0.517 |
| **Gender** | 0.498 | 0.510 |
| **Grade** | 0.654 | 0.610 |
| **Histology** | 0.592 | 0.631 |
| **IDH1 status** | 0.723 | 0.600 |
| **HRD** | 0.536 | NA |
| **Risk score** | 0.873 | 0.716 |

NA, no data.


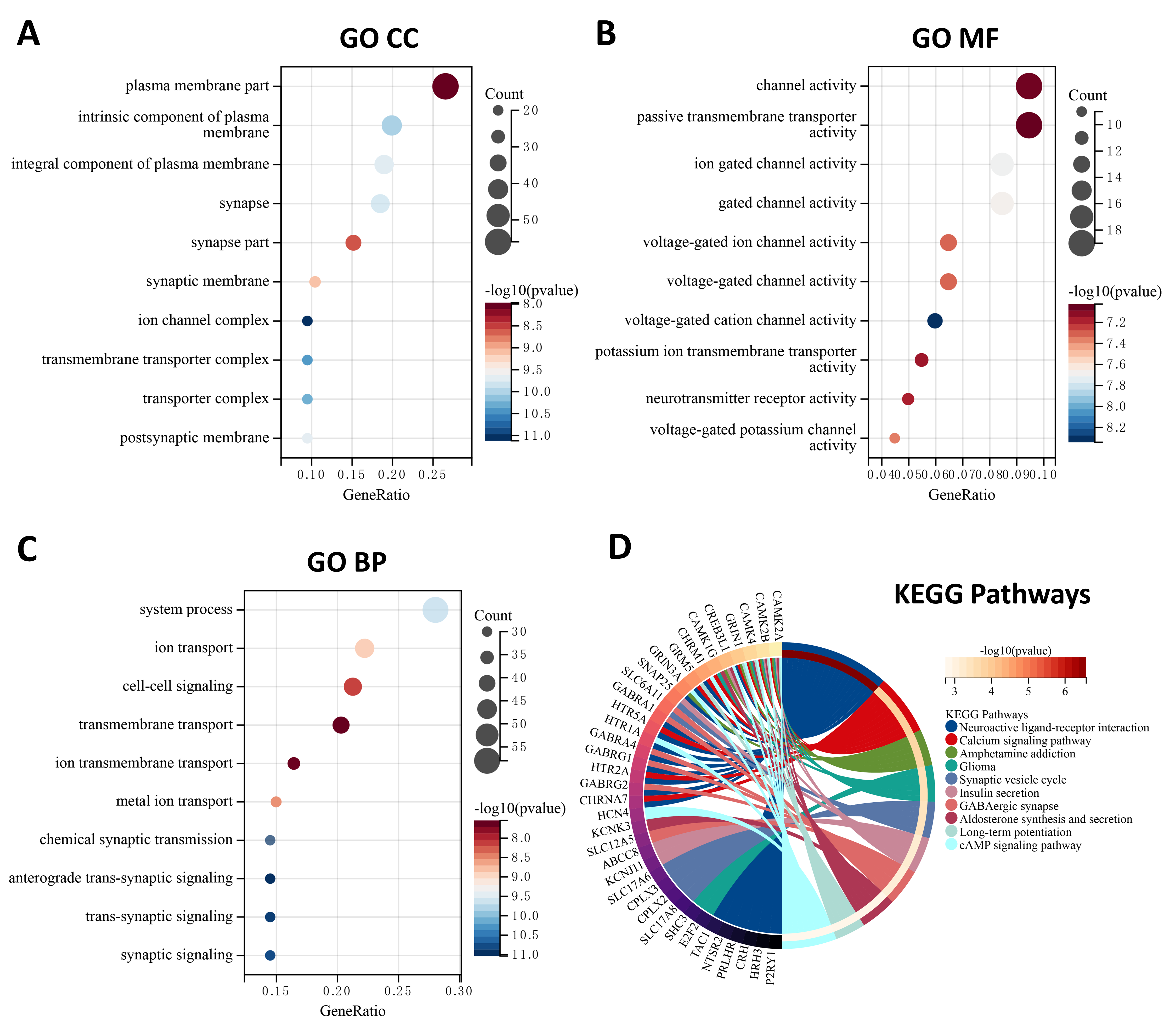


**Supplementary Figure S1. GO enrichment analysis and KEGG pathway analysis of HRD-related genes. A**, GO cellular component terms enriched in HRD-related genes. **B,** GO molecular function terms enriched in HRD-related genes. **C**, GO biological process terms enriched in HRD-related genes. **D**, KEGG pathway terms enriched in HRD-related genes.


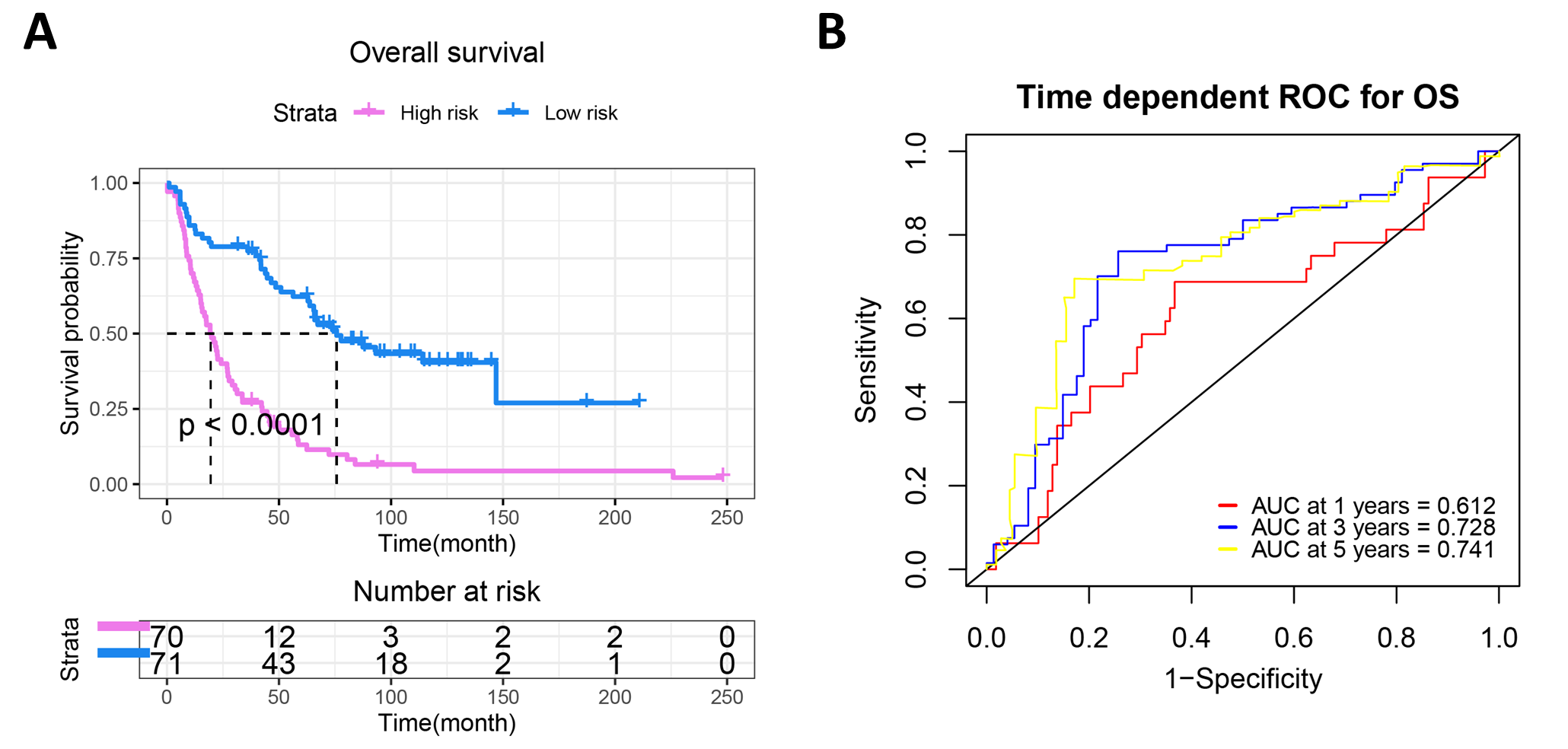


**Supplementary Figure S2.** **Prognostic predictive role of risk score in the Rembrandt cohort. A.** Kaplan-Meier curve depicts the survival difference between high-risk and low-risk groups (log-rank P<0.0001) in the CGGA cohort. Red representing the high-risk group and blue representing the low-risk group. **B.** ROC curve analysis of the risk score in the Rembrandt cohort.


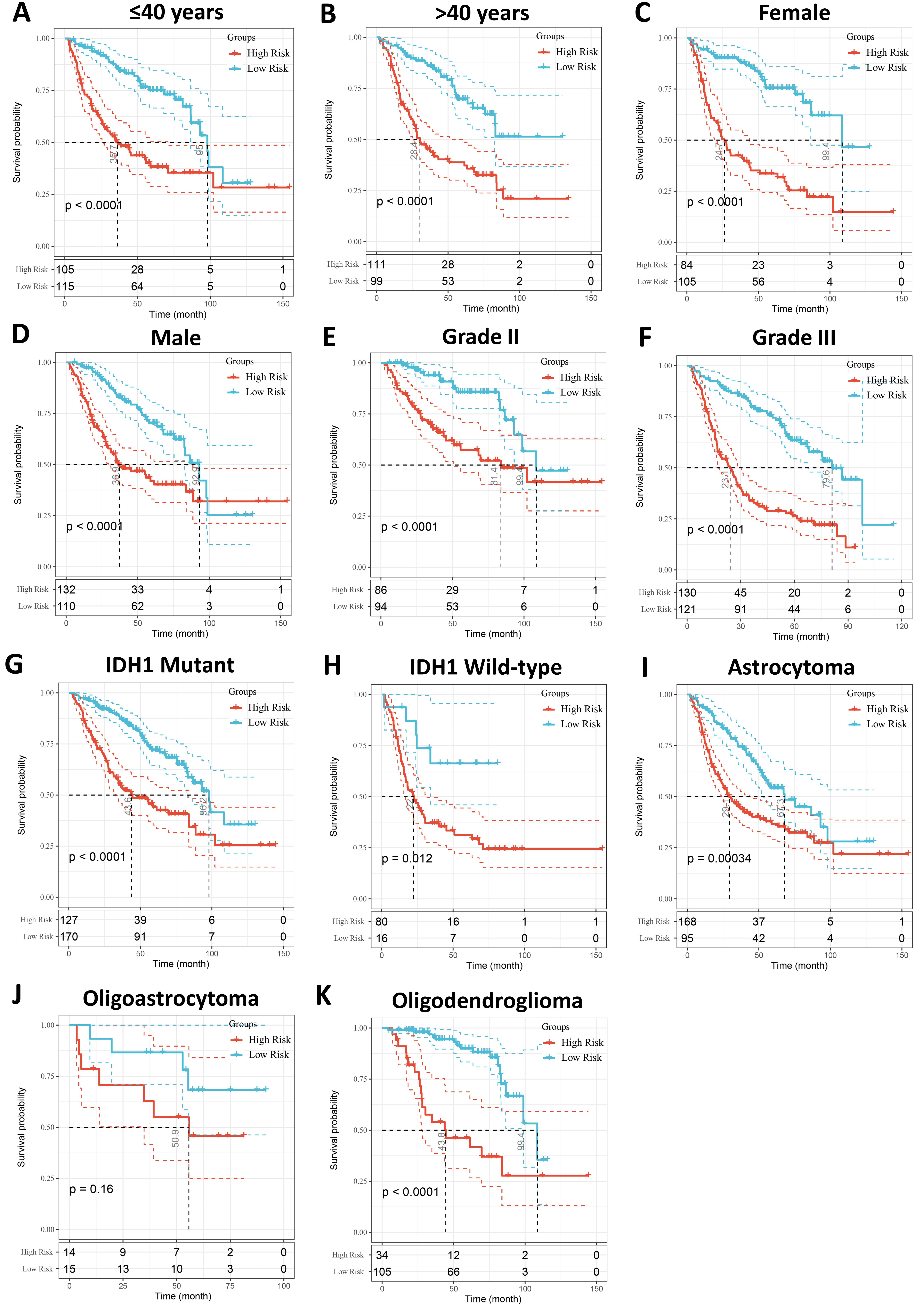


**Supplementary Figure S3. Stratified OS analysis based on the risk model in the CCGA cohort.** Based on the risk score model, stratified OS analysis performed in patients with different clinical parameters, such as age (**A and B**), gender (**C and D**), WHO grade (**E and F**), IDH1 status (**G and H**) and histological type (**I-K**) in the CCGA cohort. Significance for survival analysis was calculated using a log-rank test, with the red line representing the high-risk group and the blue line representing the low-risk group. The grouping of LGG samples is shown at the bottom of the charts.


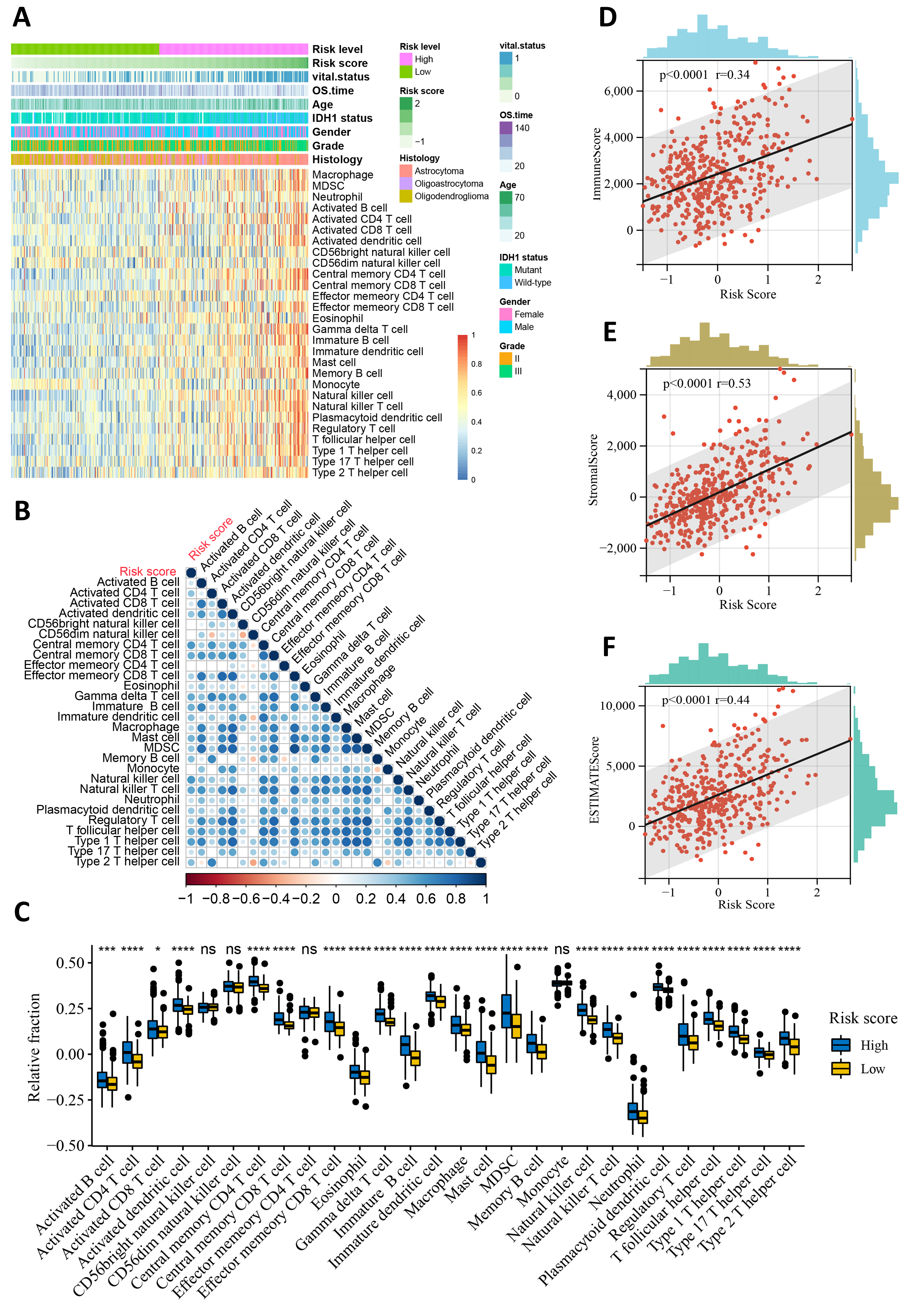


**Supplementary Figure S4. Relationship between risk score and immune cell infiltration in the CCGA cohort. A,** Heatmap of the relationship between risk score and 28 immune cells in the CCGA cohort. Age, IDH1 status, gender, vital status, OS time, histologic subtype and WHO grade are shown as patient annotations. **B,** Correlations of risk score with abundance of 28 immune cells. Correlation coefficients are calculated by Spearman’s correlation analysis, with red representing negative correlations and blue representing positive correlations. **C,** Boxplots of the relationship between risk score and 28 immune cells. The upper and lower ends of the boxes represented interquartile range of values. The lines in the boxes represented median value, and black dots showed outliers. **D,** Positive correlation between the ImmuneScore and risk score (Spearman's rank correlation coefficient, r = 0.34, P < 0.0001). **E,** Positive correlation between the StromalScore and risk score (Spearman's rank correlation coefficient, r = 0.53, P < 0.0001). F, Positive correlation between the EstimateScore and risk score (Spearman's rank correlation coefficient, r = 0.44, P < 0.0001).


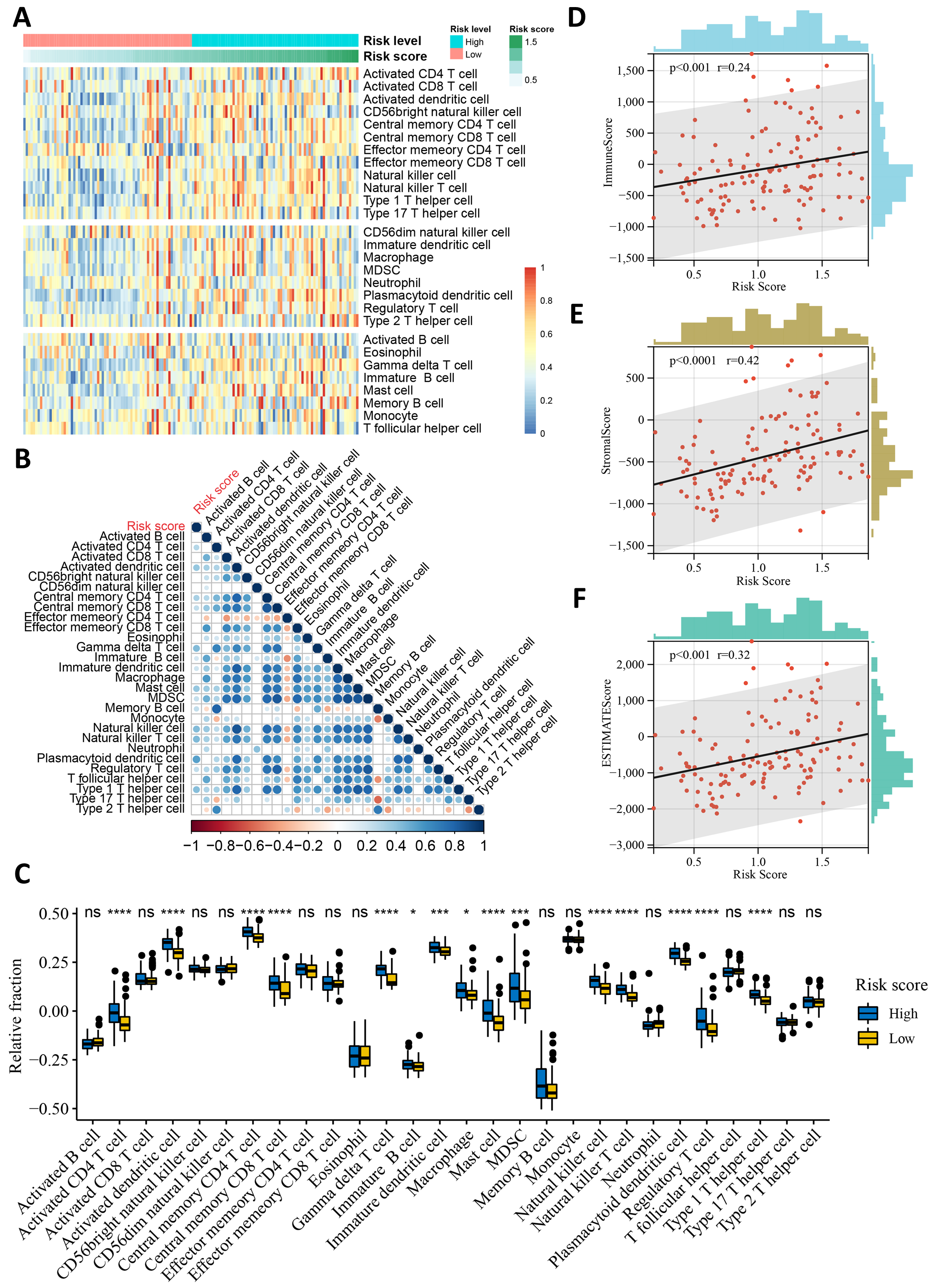


**Supplementary Figure S5. Relationship between risk score and immune cell infiltration in the Rembrandt cohort. A,** Heatmap of the relationship between risk score and 28 immune cells in the Rembrandt cohort. **B,** Correlations of risk score with abundance of 28 immune cells. Correlation coefficients are calculated by Spearman’s correlation analysis, with red representing negative correlations and blue representing positive correlations. **C,** Boxplots of the relationship between risk score and 28 immune cells. The upper and lower ends of the boxes represented interquartile range of values. The lines in the boxes represented median value, and black dots showed outliers. **D,** Positive correlation between the ImmuneScore and risk score (Spearman's rank correlation coefficient, r = 0.24, P < 0.001). **E,** Positive correlation between the StromalScore and risk score (Spearman's rank correlation coefficient, r = 0.42, P < 0.0001). F, Positive correlation between the EstimateScore and risk score (Spearman's rank correlation coefficient, r = 0.32, P < 0.001).
